# Running‐wheel activity delays mitochondrial respiratory flux decline in aging mouse muscle via a post‐transcriptional mechanism

**DOI:** 10.1111/acel.12700

**Published:** 2017-11-09

**Authors:** Sarah Stolle, Jolita Ciapaite, Aaffien C. Reijne, Alzbeta Talarovicova, Justina C. Wolters, Raúl Aguirre‐Gamboa, Pieter van der Vlies, Kim de Lange, Pieter B. Neerincx, Gerben van der Vries, Patrick Deelen, Morris A. Swertz, Yang Li, Rainer Bischoff, Hjalmar P. Permentier, Peter L. Horvatovitch, Albert K. Groen, Gertjan van Dijk, Dirk‐Jan Reijngoud, Barbara M. Bakker

**Affiliations:** ^1^ Section Systems Medicine of Metabolism and Signaling Laboratory of Pediatrics University of Groningen University Medical Center Groningen Groningen The Netherlands; ^2^ Systems Biology Centre for Energy Metabolism and Ageing University of Groningen Groningen The Netherlands; ^3^ Department of Behavioral Neuroscience Groningen Institute for Evolutionary Life Sciences (GELIFES) University of Groningen Groningen The Netherlands; ^4^ Department of Pharmacy, Analytical Biochemistry University of Groningen Groningen The Netherlands; ^5^ Department of Genetics University of Groningen University Medical Center Groningen Groningen The Netherlands; ^6^ Genomics Coordination Center University of Groningen University Medical Center Groningen Groningen The Netherlands; ^7^ Department of Vascular Medicine Amsterdam Medical Center Amsterdam The Netherlands; ^8^ Centre for Isotope Research University of Groningen Groningen The Netherlands

**Keywords:** integrative data analysis, metabolism, mitochondrial function, Regulation Analysis, skeletal muscle aging, targeted proteomics

## Abstract

Loss of mitochondrial respiratory flux is a hallmark of skeletal muscle aging, contributing to a progressive decline of muscle strength. Endurance exercise alleviates the decrease in respiratory flux, both in humans and in rodents. Here, we dissect the underlying mechanism of mitochondrial flux decline by integrated analysis of the molecular network.

Mice were given a lifelong ad libitum low‐fat or high‐fat sucrose diet and were further divided into sedentary and running‐wheel groups. At 6, 12, 18 and 24 months, muscle weight, triglyceride content and mitochondrial respiratory flux were analysed. Subsequently, transcriptome was measured by RNA‐Seq and proteome by targeted LC‐MS/MS analysis with ^13^C‐labelled standards. In the sedentary groups, mitochondrial respiratory flux declined with age. Voluntary running protected the mitochondrial respiratory flux until 18 months of age. Beyond this time point, all groups converged. Regulation Analysis of flux, proteome and transcriptome showed that the decline of flux was equally regulated at the proteomic and at the metabolic level, while regulation at the transcriptional level was marginal. Proteomic regulation was most prominent at the beginning and at the end of the pathway, namely at the pyruvate dehydrogenase complex and at the synthesis and transport of ATP. Further proteomic regulation was scattered across the entire pathway, revealing an effective multisite regulation. Finally, reactions regulated at the protein level were highly overlapping between the four experimental groups, suggesting a common, post‐transcriptional mechanism of muscle aging.

## 1 INTRODUCTION

Human aging is accompanied by a decline of skeletal muscle mass and strength, phenomena called sarcopenia and dynapenia, respectively (Manini & Clark, 2012; Rosenberg, 1997). It is estimated that up to 33% of elderly people suffer from age‐related muscle loss, the exact number depending on age and regional variation (Cruz‐Jentoft et al., 2014). Loss of muscle mass and strength can eventually lead to frailty and reduced quality of life in elderly people. The most effective strategy to slow down muscle decline is physical exercise. More specifically, resistance training, characterized by short‐duration and high‐intensity bouts, is the most effective way to counteract loss of muscle mass (Cartee, Hepple, Bamman, & Zierath, 2016; Cruz‐Jentoft et al., 2014; Joseph, Adhihetty, & Leeuwenburgh, 2015). The loss of muscle strength, however, proceeds much faster than the loss of muscle mass (Goodpaster et al., 2006). This is in part attributed to neuromuscular coupling (Manini & Clark, 2012) and in part to intrinsic muscle characteristics such as intramuscular fat accumulation (Delmonico et al., 2009; Goodpaster et al., 2001; Marcus et al., 2012; Rivas et al., 2016) and mitochondrial function (Gouspillou, Bourdel‐Marchasson, et al. 2014; Hepple, 2016; Short et al., 2005).

Here, we focus on the role of mitochondrial respiratory flux in the aging skeletal muscle. Mitochondria play a pleiotropic role in decline of muscle quality. First, reduced mitochondrial DNA and protein content have been associated with loss of muscle strength in the elderly (Short et al., 2005). Second, the generation of reactive oxygen species and opening of the mitochondrial permeability transition pore, the latter leading to apoptosis, play an important role (Chabi et al., 2008; Gouspillou, Sgarioto, et al. 2014). Last but not least, the capacity of mitochondria to supply the muscle with Gibbs energy in the form of ATP, by oxidation of carbohydrates and fatty acids, tends to decline with age. This role of mitochondrial respiratory capacity in muscle decline has been debated, but was recently demonstrated convincingly in vivo (Gouspillou, Bourdel‐Marchasson, et al. 2014). Different exercise protocols have been shown to attenuate this aging‐induced mitochondrial dysfunction in rodent (Kang, Chung, Diffee, & Ji, 2013; Ringholm et al., 2013) and human muscle (Konopka, Suer, Wolff, & Harber, 2014). Whereas the muscle mass is best retained by resistance training, muscle strength and mitochondrial respiratory capacity are most effectively retained by endurance training (Egan & Zierath, 2013; Joseph et al., 2015). This phenomenon is recapitulated in laboratory animals with access to a running wheel (Figueiredo et al., 2009; White et al., 2016), providing an excellent in vivo model to study the underlying mechanism.

The mitochondrial oxidative phosphorylation capacity can be regulated at different levels, including mitochondrial density, concentrations of mRNAs encoding mitochondrial enzymes, and the mitochondrial proteins and metabolites themselves. mRNA abundances have been measured in biopsies from young and elderly people (Irving et al., 2015), with and without regular exercise regimes (Johnson, Lanza, Short, Asmann, & Nair, 2014). For aging rodents, multiple muscle proteome and transcriptome profiles are available, measured at different time points and subject to different interventions (Alves et al., 2010; Hwang et al., 2014; Ibebunjo et al., 2012; Padrao, Ferreira, Amado, Vitorino, & Duarte, 2016). These transcriptome and proteome data were, however, analysed separately from each other and from metabolic data. To be able to disentangle how changes at the transcriptomic, proteomic and metabolomic level might affect the respiratory flux in aging skeletal muscle, it is important to make use of recent advances in quantitative proteomics technology and collect and integrate the different types of data in a single study.

**Figure 1:**
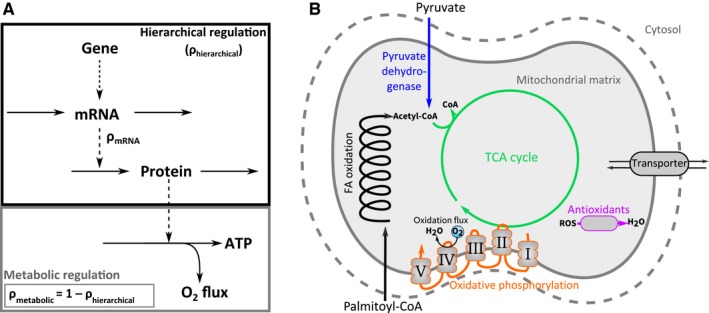
Overview of Regulation Analysis and system of interest. (a) The flow of information from gene, via mRNA, to proteins and metabolic flux. Regulation coefficients are indicated in the scheme. (b) Scheme of mitochondrial substrate oxidation. Colour coding of different pathways represented here is also used in Figure 5

Regulation Analysis (Daran‐Lapujade et al., 2007; Kuile & Westerhoff, 2001) is a quantitative methodology that integrates different levels of regulation and dissects how much each factor contributes to a change in metabolic flux. In this framework, the flux regulation upon a transition from one condition to another, for example from young to old, is first dissected into two main contributions: hierarchical and metabolic regulation (Figure 1a). Hierarchical regulation comprises the entire gene expression cascade from DNA, via mRNA to the actual enzyme concentration. Metabolic regulation integrates all metabolic effects, including the concentrations of substrates, products and allosteric effectors, as well as the affinity of the enzymes towards these metabolites, for example due to post‐translational modifications. Subsequently, hierarchical regulation can be dissected further into contributions of the involved processes (Daran‐Lapujade et al., 2007; Haanstra et al., 2008), such as translation efficiency.

The aim of this study was to disentangle in detail how the mitochondrial respiratory flux is regulated in aging skeletal muscle. We followed mice throughout their lives under four conditions: either on a low‐fat (LF) or on a high‐fat sucrose (HFS) diet, with (+) or without (−) access to a running wheel. We report a comprehensive multilevel analysis of the respiratory flux of muscle mitochondria. We show that voluntary running‐wheel activity delays the age‐related decline of mitochondrial respiratory flux irrespective of the diet. By performing a detailed Regulation Analysis, we reveal that this decline is primarily regulated at the level of protein and metabolite concentrations, while mRNA expression levels play a minor role. Once the physically active mice start losing their respiratory capacity, a full year later than the sedentary mice, all groups seem to follow largely the same mechanism.

## 2 RESULTS

### 2.1 Animal and muscle characteristics

**Figure 2:**
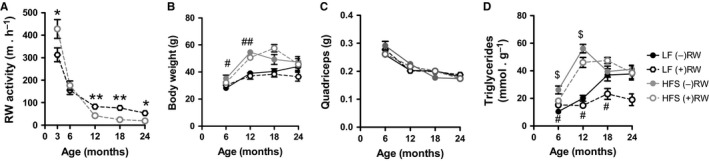
Skeletal muscle properties. (a) Running‐wheel (RW) activity. (b) Body weight. (c) Quadriceps muscle weight. (d) Triglyceride content in quadriceps muscle. Data shown as average of *n* = 7–8 mice per experimental group and time point ± SEM, except for panel A with *n* = 15–23. **p < .001 and *p < .05 compared to HFS (+)RW group; ^##^ *p* < .001 and ^#^ *p* < .05 compared to LF (−)RW group; ^$^ *p* < .05 compared to HFS (−)RW group. LF (−)RW, low‐fat without running wheel; LF (+)RW, low‐fat with running wheel; HFS (−)RW, high‐fat sucrose without running wheel; HFS (+)RW, high‐fat sucrose with running wheel

To investigate how voluntary running‐wheel activity affected mouse body weight and muscle characteristics during aging, the experimental groups were given access to a running wheel (RW). The RW activity was comparable in HFS‐ and LF‐fed mice at 6 months of age, but it was significantly lower in older HFS‐fed mice compared to LF‐fed age matched controls (Figure 2a). On both diets, the RW activity decreased with age, with the most profound drop observed between 3 and 6 months (Figure 2a). The RW activity had no effect on the body weight of mice (Figure 2b, which was confirmed by linear regression analysis of the data [*p* = .308, β  =  0.074]).

The RW activity had no effect on the loss of muscle mass during aging (Figure 2c). The mass of the hindlimb quadriceps muscle decreased 30%–40% at 24 months compared to 6 months of age in all experimental groups (Figure 2c, *p* < .001, β = 0.855). This loss was not influenced by HFS diet (*p* = .756, β = −0.015) or RW activity (*p* = .428, β = 0.039). In contrast, voluntary RW activity had a significantly negative effect on muscle triglyceride content (*p* < .01, β = −0.179), with a much more profound effect seen in LF than in HFS diet group (Figure 2d). The muscle triglyceride content was increased by both HFS diet (*p* < .001, β = 0.547) and age (*p* < .001, β = 0.386) (Figure 2d).

Upon aging, the composition of the myosin heavy chain (MHC) isoforms, which are specific for muscle fibre types (Talmadge & Roy, 1993), showed a slight shift from glycolytic (type IIb) fibres to mixed oxidative glycolytic (type IIa and IIx) fibres in the quadriceps of the LF control group (Fig. S1). In the other groups, the fibre type composition of the quadriceps muscle was stable with age.

### 2.2 Mitochondrial function and quantity

**Figure 3:**
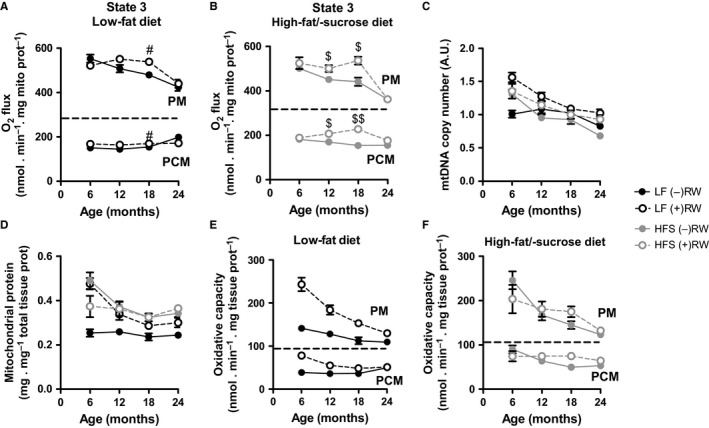
Skeletal muscle mitochondrial properties. (a, b) Maximal ADP‐stimulated O_2_ flux (state 3) in isolated skeletal muscle mitochondria oxidizing pyruvate plus malate (PM) or palmitoyl‐CoA plus L‐carnitine plus malate (PCM) in low‐fat and high‐fat sucrose diet groups. (c) Relative mtDNA copy number in quadriceps muscle. (d) Mitochondrial protein content in skeletal muscle determined as citrate synthase activity in tissue homogenate divided by that in isolated mitochondria, both normalized for the protein content of the preparation (Fig. S2C and D, Supporting information). (e, f) Maximum skeletal muscle oxygen flux capacity expressed per total tissue protein, determined as the state 3 O_2_ flux in isolated mitochondria multiplied by the mitochondrial protein content in skeletal muscle. Data are averages of *n* = 7–8 mice per experimental group and time point ± SEM. ^#^ *p* < .05 compared to LF (−)RW group; ^$$^ *p* < .001 and ^$^ *p* < .05 compared to HFS (−)RW group. Experimental group abbreviations as in Figure 2; PM, pyruvate plus malate; PCM, palmitoyl‐CoA plus L‐carnitine plus malate

Next, we turned our attention to muscle energy metabolism. The maximal ADP‐stimulated O_2_ flux (state 3) in isolated skeletal muscle mitochondria oxidizing pyruvate plus malate declined in response to HFS diet (*p* < .001, β = −0.291) and age (*p* < .001, β = 0.595), while RW activity had a significantly positive effect (Figure 3a,b and Fig. S8A, *p* < .01, β = 0.209). The comparison of O_2_ fluxes at the individual time points showed that O_2_ flux declined steadily in the sedentary LF and HFS groups, while RW activity was effective in maintaining unaltered O_2_ flux up to 18 months of age for both LF and HFS conditions (Figure 3a,b). This beneficial effect of RW activity was lost at 24 months of age (Figure 3a,b).

The mitochondrial capacity to oxidize the fatty acid substrate palmitoyl‐CoA was about a third of that of pyruvate (Figure 3a,b), as expected for muscles that largely contain glycolytic type II fibres (Fig. S1). The mitochondrial capacity to oxidize palmitoyl‐CoA was positively affected by HFS diet (*p* < .001, β = 0.285) and RW activity (*p* < .001, β = 0.351), without a significant effect of age (Figure 3a,b and Fig. S8B, *p* = .538, β = 0.049). Independently of the oxidizable substrate, the basal O_2_ flux in the absence of ATP synthesis (state 4) was somewhat negatively affected by age with no significant effect by HFS diet and a trend in the positive effect by RW activity (Fig. S2A–B).

To assess how the positive effect of RW activity on the respiratory flux of isolated mitochondria (Figure 3a,b) would affect the muscle as a whole, we estimated the muscle mitochondrial content in different ways. The relative mtDNA copy number was negatively affected by both HFS diet (*p* < .01, β = −0.168) and age (*p* < .001, β = −0.608), while RW activity had a positive effect (Figure 3c, *p* < .001, β = 0.367). The positive effect of RW activity on the mitochondrial content could be attributed to the LF diet group, as the HFS group was hardly affected by physical activity (Figure 3c,d). A similar pattern, that is a negative effect of age (*p* < .001, β = −0.324) and a positive effect of RW activity (*p* < .01, β = 0.229), was observed in the mitochondrial protein content of the muscle (Figure 3d), but the effect of HFS diet on mitochondrial protein content was inversed (*p* < .01, β = 0.412). This difference between mitochondrial DNA and protein content may be due to the fact that the estimated protein content is biased by the purity of the mitochondrial preparation (Supplementary Experimental Procedures).

To estimate the total respiratory capacity of intact muscle, we multiplied the state 3 flux of isolated mitochondria to the mitochondrial protein content of the muscle (Figure 3e,f). The total muscle oxidative flux was positively affected by HFS diet (*p* < .01, β = 0.225) and RW activity (*p* < .001, β = 0.309) and negatively affected by age (*p* < .001, β = −0.607). The positive effect of RW was very prominent in the LF diet group, because both the mitochondrial content of the muscle and the oxidative flux of isolated muscle mitochondria were stimulated by voluntary exercise. This effect was less prominent in the HFS diet group, as the positive effect of RW on the flux in isolated mitochondria was overshadowed by the positive effect of HFS diet on mitochondrial content alone.

### 2.3 Quantitative proteomics

**Figure 4:**
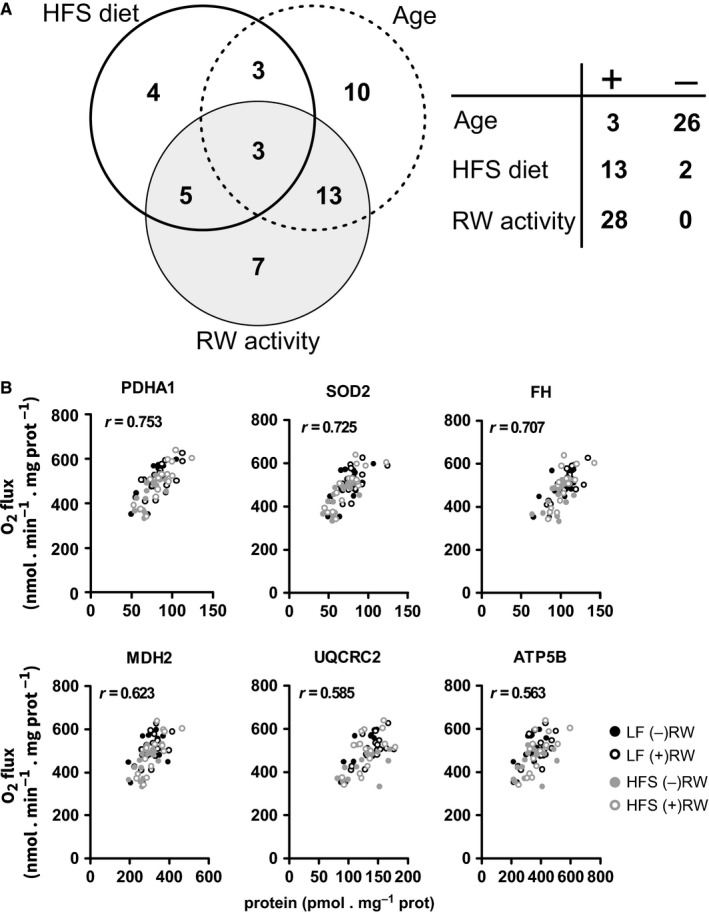
The effects of HFS diet, RW activity and age on mitochondrial proteins involved in substrate transport, OXPHOS pathway, fatty acid β‐oxidation, TCA cycle and antioxidant defence. (a) Summary of the linear regression analysis showing numbers of proteins uniquely affected by HFS diet, RW activity, and age with their overlap (Venn diagram), and direction (positive or negative) of the effect (table). (b) The correlation between the concentration of a specific mitochondrial protein and the mitochondrial O_2_ flux driven by pyruvate plus malate (PM) in state 3. All time points are combined in the same plot. Only six proteins with the highest Pearson correlation coefficient are shown. Data are *n* = 4 mice per experimental group and time point. Experimental group abbreviations as in Figure 2

To elucidate how the positive effect of exercise on the mitochondrial oxidative flux related to the underlying metabolic enzymes, we quantified the concentrations of 54 selected proteins in the mitochondria by targeted proteomics with isotopically labelled peptide standards. The selected proteins comprised the complete pathways for mitochondrial oxidation of pyruvate and palmitoyl‐CoA, including all enzymes involved in the TCA cycle and fatty acid β‐oxidation, pyruvate dehydrogenase, the relevant mitochondrial substrate transporters, several antioxidant enzymes, and a mitochondrial and nuclear encoded catalytic subunit of each of the OXPHOS complexes. A complete linear regression analysis is reported in Table S1. Age had a predominantly negative effect on the concentrations of enzymes involved in mitochondrial oxidative flux, with 26 of 29 significantly affected proteins decreasing in concentration (Figure 4a and Fig. S3). Only three proteins, the NADP‐dependent isoform of isocitrate dehydrogenase (IDH2), aconitase (ACO2) and the glutamate carrier 1 (SLC25A22), were positively affected by age. As the protein concentrations were normalized to the total protein content of the mitochondrial preparation, the decrease in the 26 downregulated proteins should be balanced by an equally increased amount of other proteins. The measured proteins are, however, only a subset of the more than 1,158 mitochondrial proteins annotated in MitoCarta2.0, and therefore, this balance does not necessarily apply within our data set. The HFS diet resulted in significant changes in the concentrations of 15 proteins with 13 proteins increasing in concentration, of which the majority (eight proteins) were involved in fatty acid β‐oxidation (Figure 4a and Fig. S3). The concentrations of two proteins, IDH2 and superoxide dismutase 2 (SOD2, a mitochondrial antioxidant enzyme), decreased in response to HFS diet. RW activity had only positive effects, resulting in significant increases in the concentrations of 28 proteins in the mitochondrial fraction constituting a mix of proteins belonging to all analysed pathways (Fig. S3). The seven proteins that were exclusively affected by RW activity included enzymes involved in fatty acid oxidation (ACAA2, ETFB and SLC25A20), the TCA cycle enzyme 2‐oxoglutarate dehydrogenase (OGDH), the catalytic subunit of OXPHOS complex I (NDUFS1), and the adenine nucleotide translocase 1 (SLC25A4) and 2 (SLC25A5) isoforms, which are transporters catalyzing the exchange of mitochondrial ATP for cytosolic ADP. This suggests that OXPHOS complex I and adenine nucleotide translocases were important for RW activity‐induced preservation of mitochondrial pyruvate oxidation flux during aging (Figure 3a). Interestingly, SOD2 was one of the three proteins affected by all three factors (negatively by HFS diet and age, and positively by RW activity), suggesting alterations in mitochondrial metabolism of reactive oxygen species (ROS).

To get a first impression as to how these enzyme concentrations contributed to the specific patterns of changes in maximal O_2_ flux during aging, we correlated the concentrations of all 54 measured proteins with the O_2_ flux in state 3 with either pyruvate or palmitoyl‐CoA as the substrate (Table S2). The analysis of the pyruvate‐driven O_2_ flux yielded a large number of significant correlations with enzymes involved in the TCA cycle, the OXPHOS pathway and the antioxidant defence (Table S2 and Figure 4b), with pyruvate dehydrogenase subunit α (PDHA1) showing the strongest correlation (Pearson correlation *r* = .753, *p* < .0001). Interestingly, SOD2 showed the second strongest positive correlation with O_2_ flux (Figure 4b, Pearson correlation *r* = .725, *p* < .0001), again suggesting the importance of superoxide anion scavenging. The analogous analysis of the palmitoyl‐CoA‐driven O_2_ flux showed that the strongest correlations were observed with enzymes involved in fatty acid β‐oxidation (Table S2 and Fig. S4). Together, this suggests that the substrate‐specific enzymes (pyruvate dehydrogenase for pyruvate oxidation and the β‐oxidation enzymes for palmitoyl‐CoA oxidation) are crucial for the observed flux patterns during aging.

### 2.4 Regulation Analysis

Subsequently, we used Regulation Analysis (Kuile & Westerhoff, 2001; Daran‐Lapujade et al., 2007; cf. Introduction) to elucidate how the age‐related decline in oxygen consumption rate in isolated muscle mitochondria was regulated in the different groups. In the sedentary groups, we looked at the gradual decline of oxidative flux between 6 and 24 months. In the (+)RW groups, we compared the 18‐ to the 24‐month cohorts, because the decline set in only after 18 months. First, we calculated the hierarchical regulation coefficient ρ_*h*_. The ρ_*h*_ quantifies, for each individual enzyme, to what extent its flux change can be attributed to a change in its protein concentration. This value is the result of the complete gene expression cascade (Figure 1a). A ρ_*h*_ value of 1 means that the rate of the enzyme can be fully explained by a change in its protein concentration. Alternatively, metabolic alterations, such as a change in substrate, product, effector concentrations or post‐translational modifications, may play a role. It can be derived (see Supplemental Experimental Procedures) that the metabolic regulation, quantified by the metabolic regulation coefficient ρ_*m*_, and the hierarchical regulation coefficient sum up to 1 (Figure 1a). The difference between this approach and Pearson correlation is that (i) Regulation Analysis reveals hierarchical regulation even when it occurs only under a subset of experimental conditions and (ii) by inference, it gives also insight into metabolic regulation. The two methods are complementary as we included all experimental groups in the Pearson correlation, while with Regulation Analysis we zoom in on a subset of the data.

**Figure 5:**
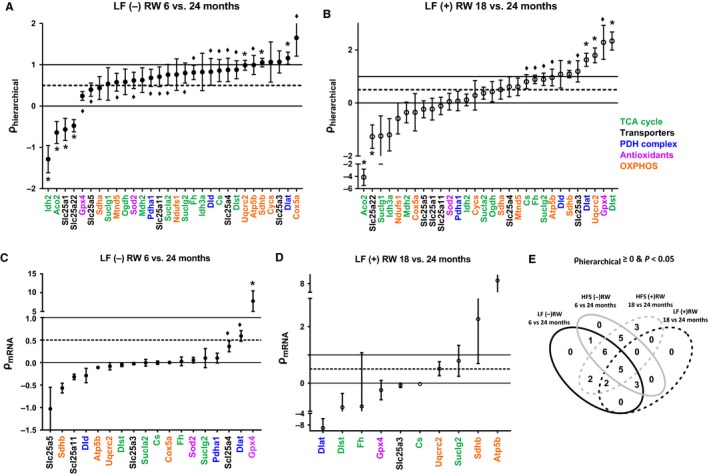
Regulation of O_2_ flux in skeletal muscle mitochondria of aging mice. (a, b) Hierarchical regulation coefficient ρ_*h*_ for LF (−) RW (a) and LF (+) RW mice (b). A coefficient of 1 means that the change in flux during aging can be explained completely by the change in protein concentration, whereas a coefficient of 0 means that the flux is completely metabolically regulated (*n* = 4 per group). (c, d) Transcriptional regulation coefficient ρ_*m*_ _*RNA*_ for LF (−) RW and LF (+) RW mice. Only proteins with a ρ_*h*_ > 0 and *p* < .05 were taken into account. The coefficients are based on *n* = 3 for mRNA,* n* = 4 for protein per group. The average ± *SD* is plotted in ascending order independently for each condition for all regulation coefficients. *(ρ_*h*_ ≠ 0.5), ♦ (ρ_*h*_ > 0) each with adjusted *p* value < .05. If the same protein was significant for *ρ_h_ ≠ 0.5 and ♦ ρ_h_ > 0, then only * was indicated. The enzymes that belong to the same metabolic pathway are highlighted in the same colour as pathways represented in Figure 1. (e) Venn diagram showing reactions with a ρ_*h*_ > 0 and *p* < .05, where regulation of protein concentration contributes significantly and often more than 50% to the change in oxidative flux with age

We calculated the hierarchical regulation coefficients of all enzymes in the pyruvate oxidation pathway and ranked them according to their value (Figure 5a,b). We found that the regulation of the O_2_ flux was shared between metabolic and hierarchical factors in all groups (Figure 5a,b and Fig. S5A–B). Most ρ_*h*_ values were between 0 (pure metabolic regulation) and 1 (pure hierarchical regulation). A substantial number of enzymes had a ρ_*h*_ value outside of these boundaries, implying that metabolic effectors and regulation of protein concentration had opposite effects on the overall enzyme rate. At ρ_*h*_ ≥ 0.5, the hierarchical regulation explains at least half of the observed change in flux. While only a small subset of enzymes showed a significantly higher ρ_*h*_ > 0.5 (between 10% and 20% depending on the group), many enzymes showed a significant hierarchical regulation component (defined as significantly larger ρ_*h*_ > 0). The HFS (+)RW group showed the highest number of proteins with a hierarchical regulation component (80%), followed by the LF (−)RW group (67%), the HFS (−)RW group (63%) and the LF (+)RW group (37%). The other enzymes (20%–63%, depending on the group) were predominantly metabolically regulated. This may be due to changes in the metabolites that directly affect these enzymes or in their affinity towards these metabolites. Of 27 proteins with a substantial hierarchical regulation 16 were shared between three or four conditions (Figure 5e and Table S3). Hierarchically regulated enzymes, however, did not cluster in any predefined metabolic pathways as can be seen from the colour coding in Figure 5a,b. For comparison, regulation coefficients were also calculated for the 6‐ vs 24‐month running‐wheel groups with similar results (Fig. S9).

Most of the enzymes that showed a significant hierarchical component (ρ_*h*_ > 0, *p* < .05) also showed a predominant hierarchical regulation (ρ_*h*_ ≥ 0.5), albeit the latter not always significant. For this subset, we calculated the regulation of the protein concentration by changes in mRNA abundance. This is quantified by ρ_*mRNA*_ and calculated from the above described proteome data set as well as RNA‐Seq analysis (Fig. S7 and Table S5) of all the mouse groups. Analogous to the above, all the hierarchical regulation downstream of the mRNA concentration, such as regulation of protein translation, protein degradation and stability, equals 1—ρ_*mRNA*_ (derived mathematically in supplemental experimental procedures). Consequently, complete regulation of protein concentrations by mRNA levels would result in a ρ_*mRNA*_ value of 1, while a lower value implies a contribution by post‐transcriptional regulation. Surprisingly, for most enzymes the ρ_*mRNA*_ was below 0.5 (Figure 5c,d and Fig. S5C–D). This means that changes in mRNA abundances made only a minor contribution to regulation of protein concentrations, with incidental exceptions. The few enzymes that were transcriptionally regulated differed per condition. Overall, protein concentrations decreased up to 50% with age, with an average decrease of 24%, whereas abundance of the corresponding mRNAs increased on average by 8% (Fig. S6).

In summary, the age‐related decline in oxidative flux is mainly regulated at post‐transcriptional and metabolic levels under all tested conditions. The fact that the majority of enzymes with a significant hierarchical regulation component ρ_*h*_ were shared among at least three of the four conditions suggests a similar mechanism of decline of oxygen consumption flux for all groups, yet, delayed by 12 months in the RW cohorts.

## 3 DISCUSSION

Here, we present an integrated, multilevel analysis of the impact of voluntary exercise and dietary intervention on mitochondrial respiratory function in skeletal muscle of aging mice. We confirmed that voluntary running, a form of endurance exercise, protected mice from decline of mitochondrial respiratory capacity until 18 months of age. We furthermore showed that this was independent of the administered diet. To elucidate the mechanism of respiratory capacity decline, we integrated transcriptome, proteome and flux data using Regulation Analysis. We found that in all groups, the decline of respiratory capacity was primarily regulated at post‐transcriptional levels, including protein synthesis and degradation on the one hand and metabolic regulation on the other. Three aspects of our study design were crucial. First, the acquisition of full time courses allowed us to characterize the beneficial effect of the running wheel, which would have been lost in a simple comparison of 6‐ and 24‐month‐old mice. Second, accurate quantification of the proteome was pivotal to reach significant regulation coefficients. To this end, we recently developed the targeted proteomics methodology with ^13^C‐labelled standards (Wolters et al., 2016). Third, the combination of RW and HFS intervention is rare and allowed to dissect commonalities, differences and interactions between different diets and exercise regimes.

In this section, we will first discuss our findings in the context of the lively debate about the relative contributions of transcriptional, translational and metabolic regulation to aging and biological adaptation. Second, we will discuss the impact of our findings for skeletal muscle aging per se.

### 3.1 Dominant regulation by translation and metabolism, not by transcription

Our finding that alterations of the proteome are only to a minor extent caused by transcriptional regulation is in sharp contrast to the work of Edfors et al. (Edfors et al., 2016). These authors reported a gene‐specific linear correlation between mRNA and protein levels across a wide range of tissues, including muscle, while we showed that changes in transcript levels are minor compared to those in protein levels. Like them, we used quantitative, targeted proteomics and RNA sequencing. Thus, the methodology could not be the reason for the discrepancy. What could then be the reason? First, Edfors et al. selected 55 proteins of interest based on—among other criteria—their high degree of variability between tissues and cell lines. These individual proteins varied over multiple orders of magnitude. In the present study, and in agreement with other aging studies in rodents (Alves et al., 2010; Hwang et al., 2014; Ibebunjo et al., 2012; Ori et al., 2015; Walther & Mann, 2011) and humans (Short et al., 2005), we show relatively small changes in protein concentrations between age groups, rarely exceeding a twofold change (Table S4). Conceivably, larger changes involved in stable tissue differentiation may be regulated primarily at the transcriptome level, while more subtle metabolic adaptations are regulated downstream. This suggestion is in agreement with the results of Ori et al. (Ori et al., 2015), who showed that aging resulted in small changes of protein concentrations in liver and brain, caused to a large extent by transcript‐specific alterations of translational efficiency. Similarly, in aging yeast cells protein concentrations changed more substantially than mRNA levels (Janssens et al., 2015), suggesting that translational regulation during aging is evolutionary conserved. The importance of translational regulation is further corroborated by studies that explicitly consider the RNA fraction bound to ribosomes and find an improved correlation between protein and mRNA (Lahtvee et al., 2017). Secondly, we focused on metabolic enzymes, while Edfors et al. included proteins across all functional groups and only 13 of 55 were metabolic enzymes. In this respect, our results correspond to a targeted study in yeast in which energy metabolism was regulated at the level of proteome and metabolome, and less so at the level of the transcriptome (Daran‐Lapujade et al., 2007). One might wonder if our focus on mitochondrial proteins affected the results, as mitochondria have their own DNA and may behave somewhat independently from the rest of the cell. We expect this not to be the case, as most of the measured mitochondrial proteins are encoded by nuclear DNA (Wolters et al., 2016).

Whereas the relative role of transcriptional and translational regulation is heavily debated, the contribution of *metabolic* regulation has hardly been quantitatively explored in aging. Using Regulation Analysis, we found that it was equally important as the regulation of protein levels to explain the decline of respiration flux. We are not aware of other aging studies in which metabolic regulation has been quantified by Regulation Analysis. Metabolic regulation in microbes, however, has been extensively analysed (van Eunen, Rossell, Bouwman, Westerhoff, & Bakker, 2011) and is considered to be a large factor to explain observed flux changes. This makes sense, as metabolite concentrations are a direct readout of altered metabolic activity and therefore provide a more sensitive means of regulation than transcriptional regulation. In addition, this type of regulation  enables a rapid adaptation to changes in the intracellular environment and reduces the cost of protein synthesis. We need to emphasize that in this analysis, metabolic regulation includes all regulation that affects enzyme turnover rates in vivo, including post‐translational modifications, in vivo metabolite concentrations and protein damage. Indeed, oxidative protein damage in the form of carbonylation has been shown to be exacerbated in old skeletal muscle and alleviated by physical exercise (Alves et al., 2010). Also protein phosphorylation, a major regulator of catalytic activity, was found to be affected by aging in mouse brain and liver (Ori et al., 2015). Finally, we quantified the regulation in isolated mitochondria at identical substrate concentrations for all groups. In this setting, only internal metabolites in the network, such as TCA cycle intermediates and redox state, contribute to the metabolic regulation. In vivo, however, also pyruvate, palmitoyl‐CoA, ATP and ADP concentrations may vary, potentially enhancing the contribution of metabolic regulation. Indeed, major shifts in the metabolome have been found in aged skeletal muscle in vivo (Houtkooper et al., 2011). Thus, the large overall impact of metabolic regulation warrants further studies that dissect how much each of these factors individually contribute to muscle aging.

### 3.2 Exercise, aging and mitochondrial function

In agreement with earlier studies (Egan & Zierath, 2013; Joseph et al., 2015), we found that voluntary running, a type of endurance exercise, did not prevent the decline of skeletal muscle mass with age, but it did increase the mitochondrial content in the LF group as well as the mitochondrial respiratory capacity in both diet groups. The decline in mitochondrial respiratory capacity is a hallmark of muscle aging, but so far no unequivocal mechanism has explained this phenotype. We observed that voluntary running protected the mitochondrial respiratory capacity until 18 months of age. Beyond 18 months, the respiratory capacity of mitochondria isolated from running‐wheel mice declined to the level of sedentary mice. This convergence at old age may well be due to the strong decrease in their running activity. The finding that mitochondrial aging and the beneficial effect of exercise were so similar between LF and HFS diet may be more surprising, as high‐fat diets tend to accelerate aging (Solon‐Biet et al., 2014). The high degree of overlap among hierarchically regulated enzymes in different experimental groups (Figure 5e, 16 of 27 proteins shared by at least three groups), points to a common mechanism of flux decline at old age, which is only delayed in running‐wheel mice. In the following paragraphs, we analyse the identity of these proteins and find that they are mostly located at the beginning and at the end of the mitochondrial pathway.

First, when correlating the respiratory flux to the individual protein levels across all experimental groups, we found the highest correlation for substrate‐specific proteins at the entry of the mitochondrial pathways (Figure 4 and Fig. S4). Specifically, pyruvate dehydrogenase subunit PDHA1 correlated with the pyruvate oxidation flux, while multiple enzymes of the fatty acid oxidation pathway correlated with the palmitoyl‐CoA oxidation flux. As the palmitoyl‐CoA flux was only affected by the diet and not by age or exercise regime, we did not subject this flux to Regulation Analysis. With respect to the pyruvate flux, we found that all subunits of the pyruvate dehydrogenase complex (DLD, DLAT and PDHA1) were hierarchically regulated in three of four conditions (Table S3). Together, this suggests a crucial regulatory role for hierarchical regulation of this mitochondrial gatekeeper enzyme (Figure 1b) during aging. Second, hierarchically regulated proteins that were common in all four conditions (Table S3) were a subunit of the ATP synthase complex (ATP5B) and the phosphate carrier protein (SLC25A3). ATP synthase and phosphate transport are both related to the synthesis of ATP, processes at the very end of the pathway. The ATP/ADP translocator itself (SLC25A4) was hierarchically regulated in three of four conditions. Furthermore, citrate synthase (CS), the first enzyme of the tricarboxylic acid cycle, was regulated hierarchically under all four conditions. The exception was UQCRC2, a subunit of the *bc1* complex, which is located in the middle of the respiratory chain and regulated hierarchically under all conditions. We note that five of these proteins (DLAD, DLD, SLC25A4, SLC25A3 and CS) did not show a high Pearson correlation with the flux (Figure 4b), most likely because regulation is condition‐dependent and combining all data blurred the picture. This is even more relevant for proteins that contributed strongly to the hierarchical regulation under only two or three of four conditions, for example cytochrome *c* (CYCS) and the COX5a subunit of the cytochrome oxidase complex, which are both active at the very end of the respiratory chain.

A strong hierarchical regulation at the beginning and at the end of the pathway is effective, as it allows for flux regulation without major fluctuations of metabolite concentrations. Other enzymes regulated hierarchically in all but one condition were distributed over the TCA cycle and the respiratory chain. This provides a clear example of multisite regulation (Fell & Thomas, 1995), further dampening the changes in metabolite concentrations.

In addition to the hierarchical regulation, the metabolic regulation (including protein modification, protein oxidative damage and altered metabolite concentrations) contributed strongly to the respiratory flux decline. A protein that showed dominant metabolic regulation (ρ_hierarchical_ < 0.5; *p* < .05) in all four experimental groups was the TCA cycle enzyme aconitase 2 (ACO2). ACO2 is highly susceptible to oxidative damage and other post‐translational modifications due to its iron–sulphur cluster (Lushchak, Piroddi, Galli, & Lushchak, 2014). Aging increased fragmentation and protein modifications of ACO2 in rodents (Alves et al., 2010; Bota, Van Remmen, & Davies, 2002; Gannon, Staunton, O'Connell, Doran, & Ohlendieck, 2008). Additionally, the mitochondrial isocitrate dehydrogenase (IDH2) and mitochondrial glutamate transporter (SLC25A22) were predominantly metabolically regulated. SLC25A22 may not be relevant here, as we did not study the glutamate flux. IDH2 was relatively lowly expressed in our muscle samples compared to its isoenzyme IDH3A and its regulation may therefore have little impact on the overall flux.

### 3.3 Concluding remarks

In conclusion, we elucidated a crucial role for metabolic and proteomic regulation of mitochondrial respiration in aging skeletal muscle, whereas transcriptomic regulation played a minor role. The proteomic regulation was mainly found at the beginning and the end of the mitochondrial pathway, providing an effective multisite regulation.

## 4 EXPERIMENTAL PROCEDURES

Extended methods for determination of myosin heavy chain composition, triglyceride content, citrate synthase activity, mtDNA copy number, RNA isolation and analysis, statistical analysis and an extended background on Regulation Analysis can be found in Supplementary experimental procedures.

### 4.1 Animals and study design

Breeding pairs of C57BL6/JOlaHsd mice (Harlan Netherlands BV, Horst, The Netherlands) were housed on a 12‐hr:12‐hr light:dark cycle in a temperature‐controlled environment (22°C) with ad libitum access to low‐fat diet (LF, 6% calories from fat; AMII 2141, HopeFarms BV, Woerden, NL) and water. Upon weaning on day 28, male offspring were housed individually in Makrolon type II cages (Bayer, Germany) with ad libitum access to either LF diet or high‐fat sucrose diet (HFS, 45% calories from fat; 4,031.09, HopeFarms BV, Woerden, NL) and water. Each diet group was randomly subdivided into sedentary (LF (−)RW and HFS (−)RW) and voluntary running‐wheel (RW) groups (LF (+)RW and HFS (+)RW). All mice were checked daily for health, activity and behaviour abnormalities. At the age of 6, 12, 18 and 24 months, mice (*n* = 8 per experimental group for proteomics and flux, and *n* = 3 for transcriptomics) were anesthetized with 2% isoflurane followed by decapitation. The skeletal muscles (quadriceps from one hindlimb, and gastrocnemius and tibialis anterior from both hindlimbs) of *n* = 8 mice per experimental group were quickly excised and used fresh for isolation of mitochondria. Another quadriceps muscle per animal was quickly snap‐frozen in liquid nitrogen and stored at −80°C until further biochemical analysis. The animal treatment conformed to the guidelines of the Institutional Animal Care and Use Committee of the University of Groningen and was in accordance with EC Directive 86/609/EEC for animal experiments.

### 4.2 Regulation Analysis

We performed Regulation Analysis to assess to what extent hierarchical and metabolic regulation contributed to the change in respiratory capacity of skeletal muscle mitochondria during aging. First, we determined the contribution of hierarchical regulation to the change in enzymatic flux. For each enzyme *i*, we calculated the hierarchical regulation coefficients (ρ_*h, i*_) as follows:

$$\rho_{h,i}=\frac{\text{ln}e_{i,age1}-\text{ln}e_{i,age2}}{\text{ln}v_{i,age1}-\text{ln}v_{i,age2}}$$

where *e* is the enzyme concentration and *v* is the flux (both expressed per mitochondrial protein). We subtracted samples of 24‐month‐old mice (*age2*) from samples of younger mice (*age1*); for (−)RW condition, we used the 6 months’ time point, for (+)RW condition the 18 months’ time point. We assumed that all enzyme rates *v* were proportional to the measured oxygen flux. The expression of all values per mitochondrial protein implies that a measured increase can also be caused by decreased proteins outside the pathways of our study, or vice versa. If this were the dominant effect, we should expect proportional changes in all proteins as well as of the flux, leading to only ρ_*h*_ values of 1. In reality, we find a diversity of regulation (see Results) proving that regulatory events happen inside the pathways of interest.

Second, we determined to what extent reactions that showed a significant hierarchical regulation (ρ_h_ > 0; *p* < .05) were regulated at the level of mRNA concentrations. The transcriptional regulation coefficients (ρ_mRNA,i_) were calculated as follows:

$$\rho_{mRNA,i}=\frac{\text{ln}{\left[mRNA\right]}_{i,age1}-\text{ln}{\left[mRNA\right]}_{i,age2}}{\text{ln}{\left[e_{tissue}\right]}_{i,age1}-\text{ln}{\left[e_{tissue}\right]}_{i,age2}}$$

We used the proteomics data mentioned above and the mRNA abundance measured by RNA‐Seq. As the mRNA abundance was measured at the tissue level, we transformed the protein concentrations also to the tissue level by multiplying the abundance of the protein in the mitochondrial fraction with the mitochondrial protein content relative to total cellular protein content. The latter was calculated as a ratio of citrate synthase (CS) activity in the total tissue to the CS activity in mitochondrial fraction, both expressed per protein in the respective fractions. This conversion should be independent of the purity of the mitochondrial preparation, as the enrichment factor for CS is the same as for other proteins in the mitochondrial preparation. This approach does not, however, take into account any mitochondrial inhomogeneity, for example due to preferential isolation of subsarcolemmal over intermyofibrillar mitochondria (Ferreira et al., 2010).

## AUTHOR CONTRIBUTIONS

SS, JC and BMB wrote manuscript. JC, SS and ACR analysed the data. ACR, JC and AT performed animal experiments. JCW measured proteomics, under supervision of RB and HPP. JC and PLH analysed proteomics data. PV and KL measured mRNA data. PBN, GV, PD and MAS developed RNA‐Seq analysis pipeline. RAG and YL analysed RNA‐Seq data. JC, ACR, AKG, GD, DJR and BMB designed the experiments.

## CONFLICT OF INTEREST

None declared.
