# Supplementary material for: Running‐wheel activity delays mitochondrial respiratory flux decline in aging mouse muscle via a post‐transcriptional mechanism
Source: Aging Cell. 2017 Nov 9;17(1):e12700. doi: 10.1111/acel.12700 (PMC5770778; doi:10.1111/acel.12700)
Supplement: Supplementary file 1 [file ACEL-17-na-s001.pdf]

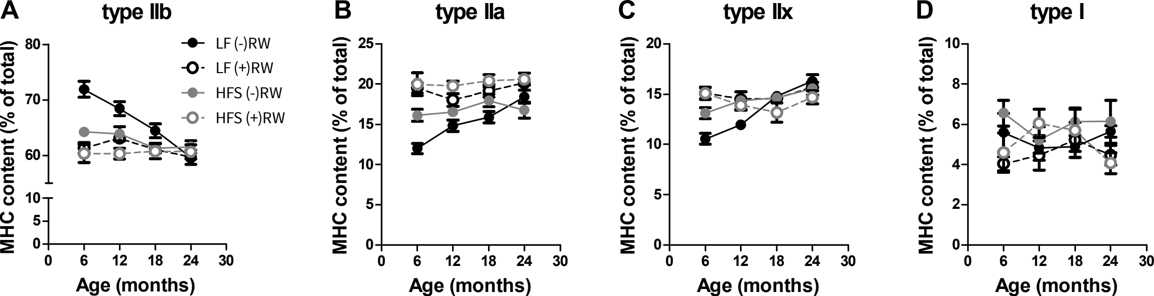

A
type IIb
B
type IIa
C
type IIx
D
type I
LF (-)RW
LF (+)RW
HFS (-)RW
HFS (+)RW
MHC content (% of total)
Age (months)
