# Supplementary figures and images for: Running‐wheel activity delays mitochondrial respiratory flux decline in aging mouse muscle via a post‐transcriptional mechanism
Source: Aging Cell. 2017 Nov 9;17(1):e12700. doi: 10.1111/acel.12700 (PMC5770778; doi:10.1111/acel.12700)

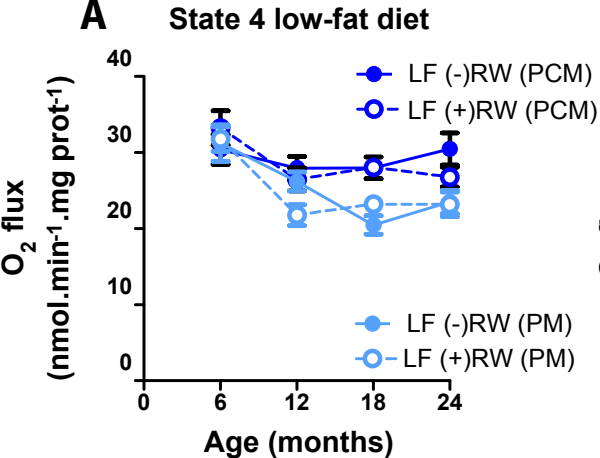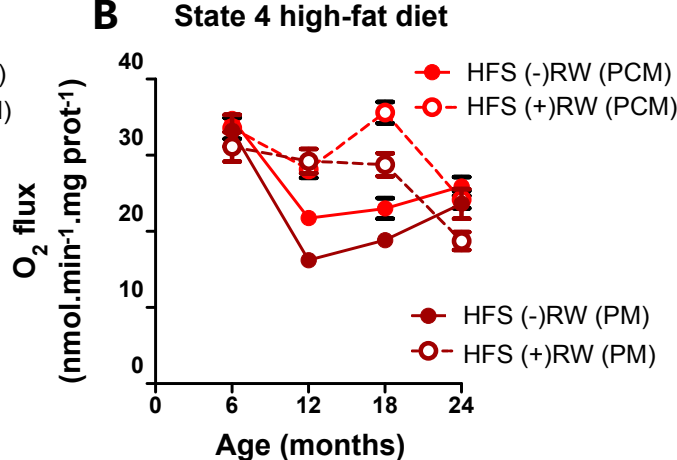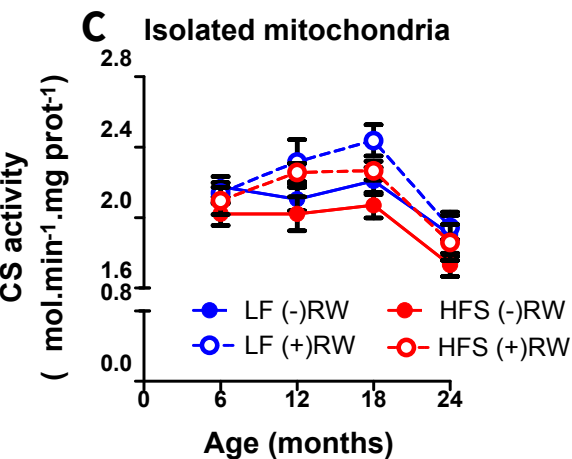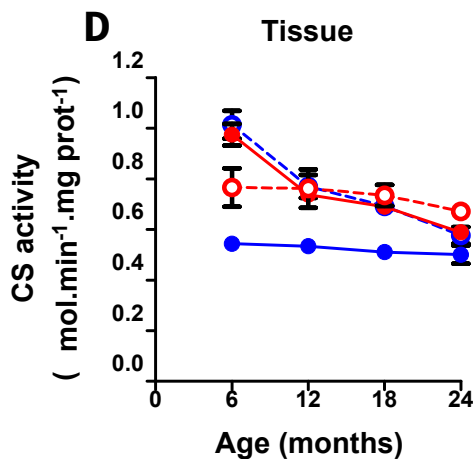

Supplement: Supplementary file 2 [file ACEL-17-na-s002.pdf]

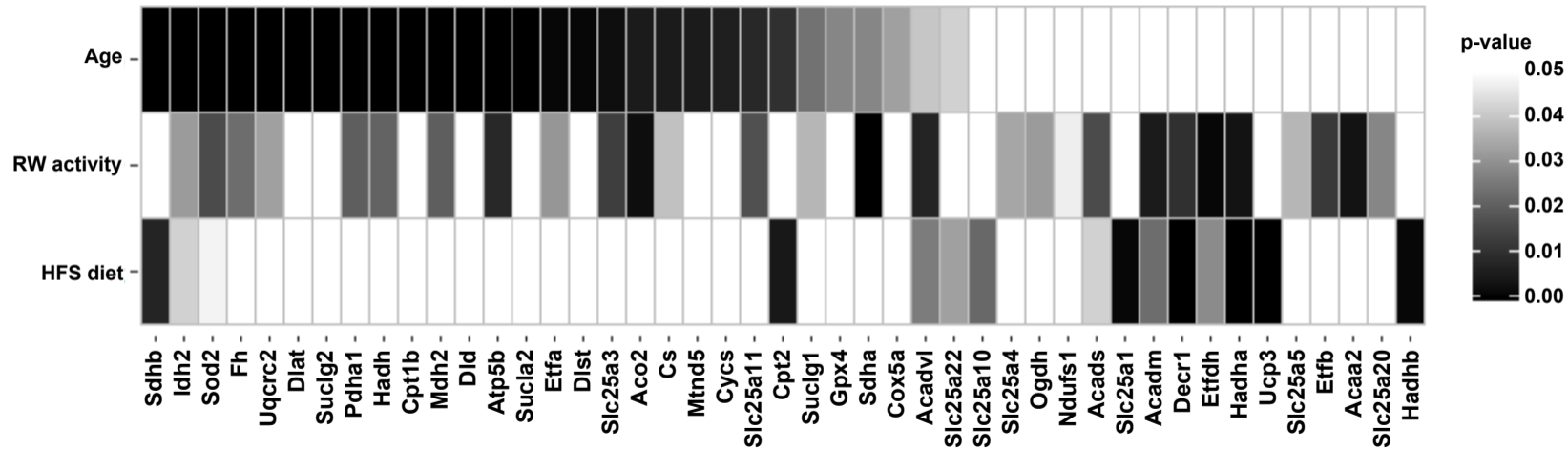

Supplement: Supplementary file 3 [file ACEL-17-na-s003.pdf]

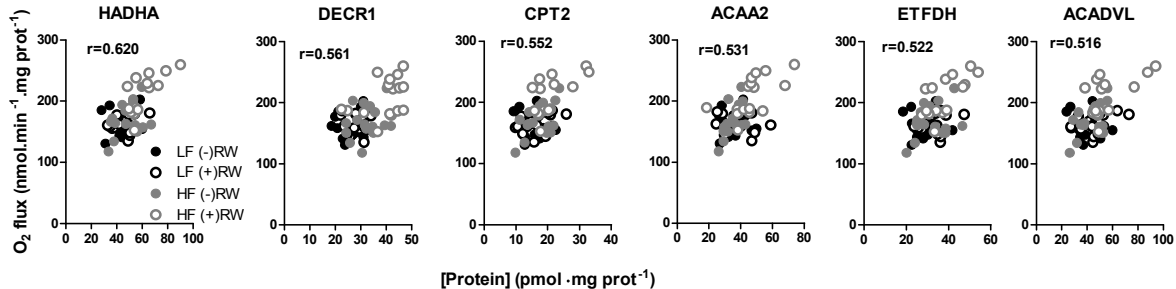

Supplement: Supplementary file 4 [file ACEL-17-na-s004.pdf]

**A**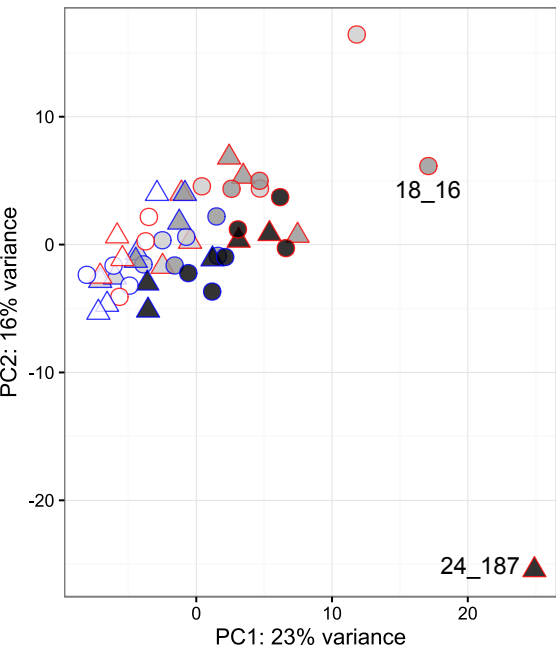**B**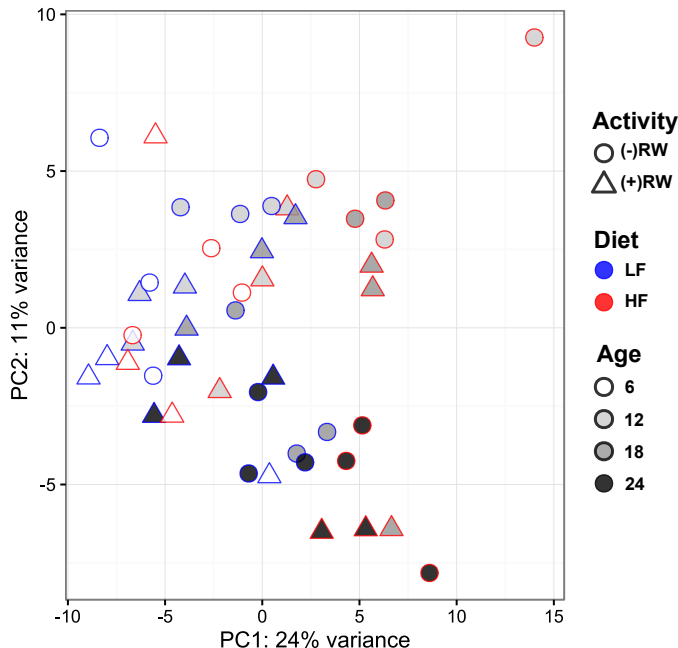

Supplement: Supplementary file 6 [file ACEL-17-na-s006.pdf]

% change between 6 and 24 months

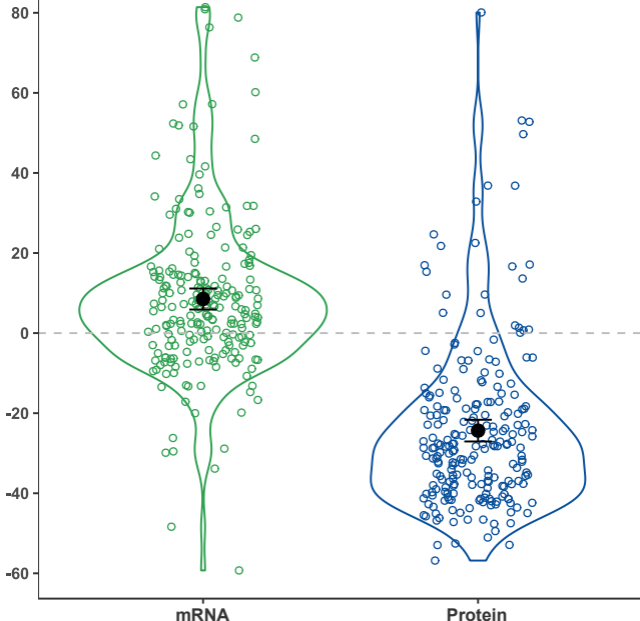

Supplement: Supplementary file 7 [file ACEL-17-na-s007.pdf]

**A**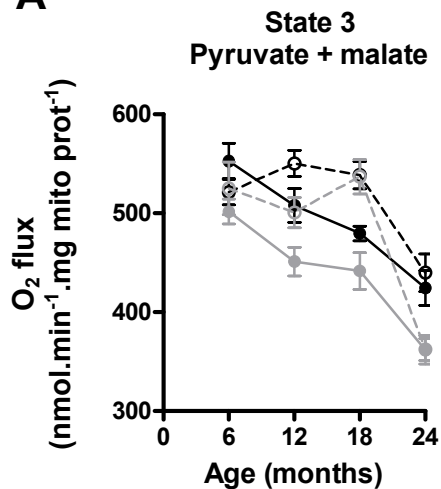**B**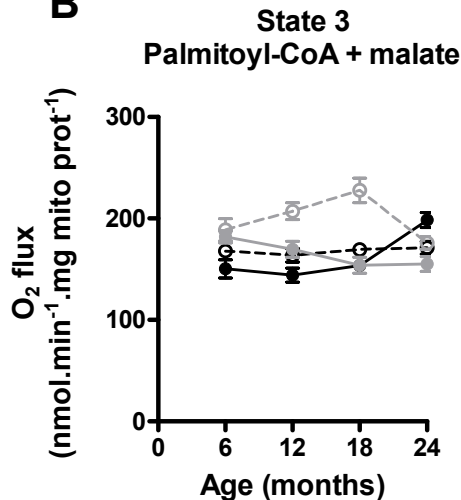**C**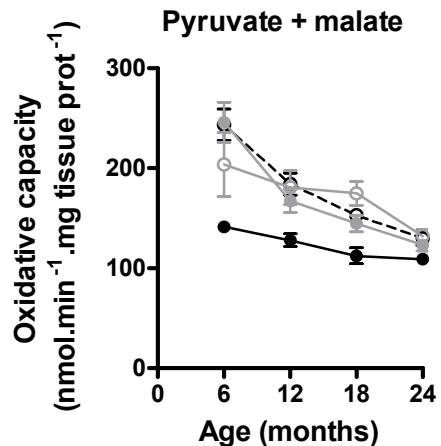**D**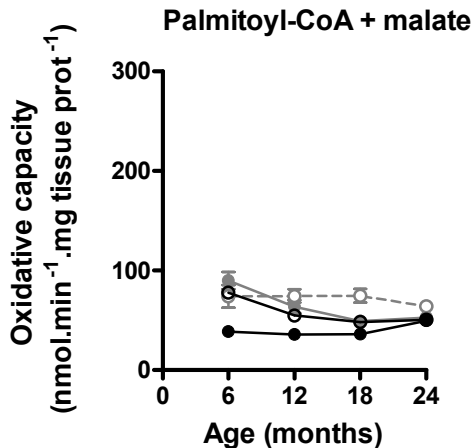

- LF (-)RW
- LF (+)RW
- HFS (-)RW
- HFS (+)RW

Supplement: Supplementary file 8 [file ACEL-17-na-s008.pdf]

**A** LF (+)RW 6 vs 24 months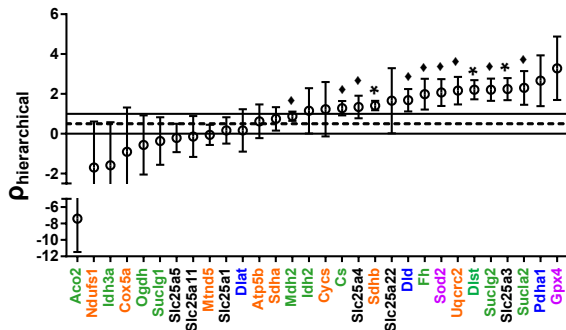**B** HFS (+)RW 6 vs 24 months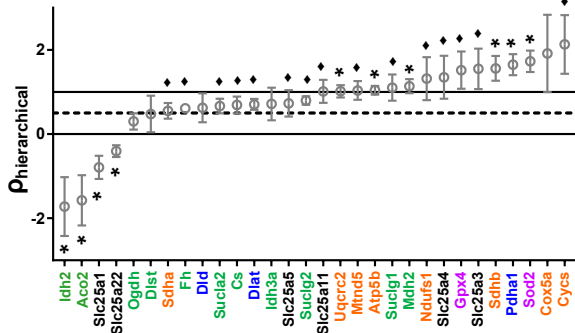**C** LF (+)RW 6 vs 24 months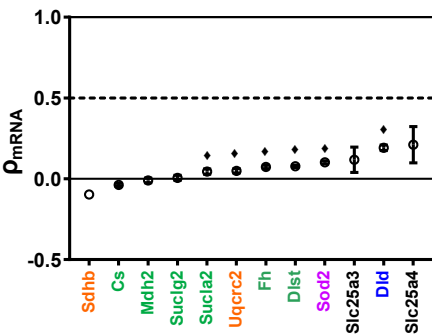**D** HFS (+)RW 6 vs 24 months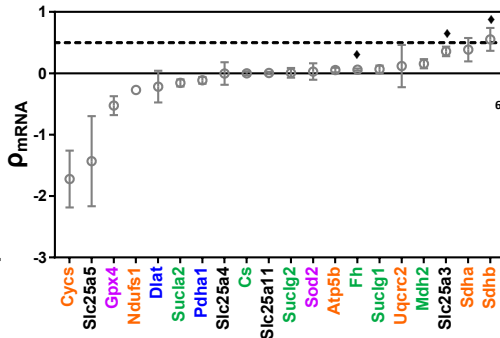**E**  $\rho_{\text{hierarchical}} \geq 0$  &  $p < 0.05$ 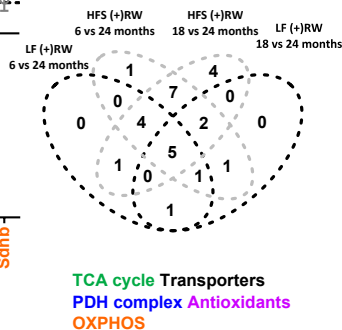

Supplement: Supplementary file 9 [file ACEL-17-na-s009.pdf]
