# Supplementary material for: Running‐wheel activity delays mitochondrial respiratory flux decline in aging mouse muscle via a post‐transcriptional mechanism
Source: Aging Cell. 2017 Nov 9;17(1):e12700. doi: 10.1111/acel.12700 (PMC5770778; doi:10.1111/acel.12700)
Supplement: Supplementary file 5 [file ACEL-17-na-s005.pdf]

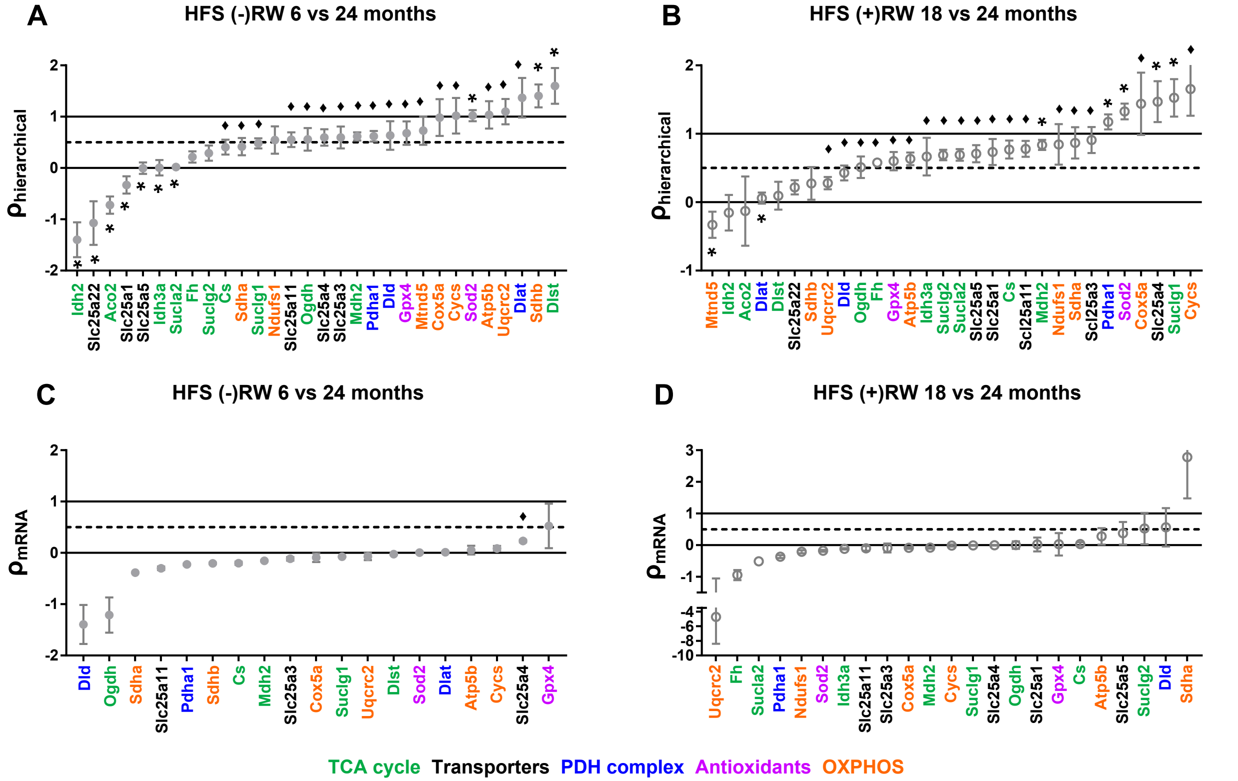
A
HFS (-)RW 6 vs 24 months
ρhierarchical
Idh2
Slc25a22
Aco2
Slc25a1
Slc25a5
Idh3a
Sucla2
Fh
Suclg2
Cs
Sdha
Suclg1
Ndufs1
Slc25a11
Ogdh
Slc25a4
Slc25a3
Mdh2
Pdha1
Dld
Gpx4
Mtnd5
Cox5a
Cycs
Sod2
Atp5b
Uqcrc2
Dlat
Sdhb
Dlst
B
HFS (+)RW 18 vs 24 months
ρhierarchical
Mtnd5
Idh2
Aco2
Dlat
Dlst
Slc25a22
Sdhb
Uqcrc2
Dld
Ogdh
Fh
Gpx4
Atp5b
Idh3a
Suclg2
Sucla2
Slc25a5
Slc25a1
Cs
Scl25a11
Mdh2
Ndufs1
Sdha
Scl25a3
Pdha1
Sod2
Cox5a
Slc25a4
Suclg1
Cycs
C
HFS (-)RW 6 vs 24 months
ρmRNA
Dld
Ogdh
Sdha
Slc25a11
Pdha1
Sdhb
Cs
Mdh2
Slc25a3
Cox5a
Suclg1
Uqcrc2
Dlst
Sod2
Dlat
Atp5b
Cycs
Slc25a4
Gpx4
D
HFS (+)RW 18 vs 24 months
ρmRNA
Uqcrc2
Fh
Sucla2
Pdha1
Ndufs1
Sod2
Idh3a
Slc25a11
Slc25a3
Cox5a
Mdh2
Cycs
Suclg1
Slc25a4
Ogdh
Slc25a1
Gpx4
Cs
Atp5b
Slc25a5
Suclg2
Dld
Sdha
TCA cycle Transporters PDH complex Antioxidants OXPHOS
