# Supplementary material for: Running‐wheel activity delays mitochondrial respiratory flux decline in aging mouse muscle via a post‐transcriptional mechanism
Source: Aging Cell. 2017 Nov 9;17(1):e12700. doi: 10.1111/acel.12700 (PMC5770778; doi:10.1111/acel.12700)
Supplement: Supplementary file 12 [file ACEL-17-na-s012.docx]

**Supplemental data**

**Supplemental Tables**

**Table S1. Linear regression analysis of targeted quantitative proteomics of isolated mitochondria.** The p values and standardized coefficients β for high-fat sucrose (HFS) diet, voluntary running wheel (RW) activity, and age for 54 quantified proteins are shown. Proteins are ranked according to the p value of the age effect.

|  |  | **p value** | | | **Standardized coefficient β** | | |
| --- | --- | --- | --- | --- | --- | --- | --- |
| # | Protein | HFS diet | RW activity | Age | HFS diet | RW activity | Age |
| 1 | UQCRC2 | 0.253 | 0.033 | 0.000 | -0.120 | 0.227 | -0.529 |
| 2 | PDHA1 | 0.485 | 0.020 | 0.000 | -0.074 | 0.252 | -0.517 |
| 3 | CPT1B | 0.594 | 0.066 | 0.000 | 0.057 | 0.200 | -0.518 |
| 4 | HADH | 0.557 | 0.021 | 0.000 | 0.063 | 0.254 | -0.499 |
| 5 | SOD2 | 0.048 | 0.016 | 0.000 | -0.212 | 0.260 | -0.472 |
| 6 | FH | 0.154 | 0.023 | 0.000 | -0.155 | 0.251 | -0.472 |
| 7 | SUCLG2 | 0.354 | 0.760 | 0.000 | 0.105 | 0.034 | -0.483 |
| 8 | SDHB | 0.007 | 0.458 | 0.000 | -0.301 | 0.080 | -0.458 |
| 9 | MDH2 | 0.603 | 0.020 | 0.000 | -0.058 | 0.262 | -0.451 |
| 10 | DLAT | 0.325 | 0.214 | 0.000 | -0.113 | 0.143 | -0.438 |
| 11 | ATP5B | 0.909 | 0.008 | 0.000 | 0.013 | 0.302 | -0.421 |
| 12 | DLD | 0.771 | 0.202 | 0.000 | 0.034 | 0.148 | -0.433 |
| 13 | SUCLA2 | 0.958 | 0.165 | 0.000 | -0.006 | 0.162 | -0.424 |
| 14 | IDH2 | 0.042 | 0.032 | 0.000 | 0.229 | 0.241 | 0.405 |
| 15 | ETFA | 0.800 | 0.031 | 0.001 | 0.029 | 0.252 | -0.398 |
| 16 | DLST | 0.988 | 0.145 | 0.001 | -0.002 | 0.171 | -0.405 |
| 17 | SLC25A3 | 0.665 | 0.013 | 0.002 | -0.049 | 0.292 | -0.376 |
| 18 | CS | 0.520 | 0.039 | 0.005 | -0.075 | 0.247 | -0.343 |
| 19 | MTND5 | 0.632 | 0.190 | 0.005 | -0.057 | 0.158 | -0.350 |
| 20 | ACO2 | 0.478 | 0.002 | 0.005 | -0.080 | 0.363 | 0.326 |
| 21 | CYCS | 0.927 | 0.314 | 0.006 | 0.011 | 0.122 | -0.342 |
| 22 | SLC25A11 | 0.461 | 0.017 | 0.008 | -0.086 | 0.286 | -0.317 |
| 23 | CPT2 | 0.004 | 0.084 | 0.010 | 0.338 | 0.198 | -0.298 |
| 24 | SUCLG1 | 0.467 | 0.037 | 0.024 | 0.087 | 0.255 | -0.275 |
| 25 | GPX4 | 0.283 | 0.277 | 0.028 | -0.132 | 0.134 | -0.275 |
| 26 | SDHA | 0.630 | 0.000 | 0.028 | -0.054 | 0.419 | -0.253 |
| 27 | COX5A | 0.377 | 0.079 | 0.033 | -0.107 | 0.215 | -0.263 |
| 28 | ACADVL | 0.026 | 0.007 | 0.040 | 0.259 | 0.316 | -0.238 |
| 29 | SLC25A22 | 0.033 | 0.220 | 0.042 | 0.260 | 0.148 | 0.247 |
| 30 | SLC25A10 | 0.022 | 0.252 | 0.063 | 0.280 | -0.138 | 0.226 |
| 31 | SLC25A4 | 0.663 | 0.034 | 0.073 | -0.053 | 0.263 | -0.221 |
| 32 | OGDH | 0.683 | 0.032 | 0.090 | -0.050 | 0.267 | -0.209 |
| 33 | NDUFS1 | 0.348 | 0.047 | 0.093 | -0.115 | 0.247 | -0.207 |
| 34 | ACADS | 0.042 | 0.016 | 0.111 | 0.243 | 0.290 | -0.189 |
| 35 | SLC25A1 | 0.001 | 0.579 | 0.112 | 0.416 | -0.064 | 0.185 |
| 36 | ACADM | 0.023 | 0.005 | 0.114 | 0.266 | 0.337 | -0.183 |
| 37 | PRDX6 | 0.707 | 0.978 | 0.141 | -0.048 | -0.004 | 0.189 |
| 38 | IDH3A | 0.861 | 0.071 | 0.143 | -0.022 | 0.227 | -0.183 |
| 39 | ECI1 | 0.191 | 0.512 | 0.190 | 0.165 | 0.082 | -0.166 |
| 40 | UCP2 | 0.345 | 0.325 | 0.201 | -0.119 | 0.124 | -0.162 |
| 41 | DECR1 | 0.000 | 0.010 | 0.302 | 0.575 | 0.263 | 0.103 |
| 42 | ETFDH | 0.029 | 0.001 | 0.319 | 0.254 | 0.382 | -0.114 |
| 43 | PDK1 | 0.495 | 0.069 | 0.364 | 0.085 | 0.230 | -0.114 |
| 44 | HADHA | 0.000 | 0.003 | 0.411 | 0.436 | 0.335 | -0.089 |
| 45 | UCP3 | 0.000 | 0.240 | 0.439 | 0.461 | 0.134 | -0.088 |
| 46 | CPT1A | 0.505 | 0.875 | 0.505 | 0.086 | 0.020 | -0.086 |
| 47 | GSR | 0.553 | 0.454 | 0.602 | -0.076 | 0.096 | 0.067 |
| 48 | SLC25A5 | 0.669 | 0.037 | 0.654 | 0.053 | 0.264 | -0.056 |
| 49 | ECHS1 | 0.933 | 0.228 | 0.703 | -0.011 | 0.155 | -0.049 |
| 50 | ETFB | 0.275 | 0.012 | 0.711 | 0.134 | 0.314 | 0.045 |
| 51 | ACAA2 | 0.073 | 0.003 | 0.768 | 0.213 | 0.368 | -0.035 |
| 52 | SLC25A20 | 0.366 | 0.028 | 0.882 | 0.157 | 0.397 | 0.026 |
| 53 | ACADL | 0.097 | 0.066 | 0.916 | 0.234 | 0.262 | -0.015 |
| 54 | HADHB | 0.001 | 0.061 | 0.919 | 0.410 | 0.218 | -0.012 |

**Table S2. Correlation between mitochondrial protein concentration and mitochondrial O_2_ flux for pyruvate plus malate (PM) and** **palmitoyl-CoA plus L-carnitine plus malate (PCM) as substrates.** The Pearson correlation coefficients and p values for each of 54 measured proteins are shown. Proteins are ranked according to their Pearson correlation coefficients for PM as the substrate. Data are n = 4 mice per experimental group and time point.

| **#** | **Protein** | **Pearson correlation (PM)** | **p-value (PM)** | **Pearson correlation (PCM)** | **p-value (PCM)** |
| --- | --- | --- | --- | --- | --- |
| 1 | PDHA1 | 0.753 | 0.0000 | 0.373 | 0.0024 |
| 2 | SOD2 | 0.725 | 0.0000 | 0.326 | 0.0086 |
| 3 | FH | 0.707 | 0.0000 | 0.298 | 0.0167 |
| 4 | CPT1B | 0.630 | 0.0000 | 0.291 | 0.0199 |
| 5 | MDH2 | 0.623 | 0.0000 | 0.417 | 0.0006 |
| 6 | UQCRC2 | 0.585 | 0.0000 | 0.115 | 0.3661 |
| 7 | ETFA | 0.576 | 0.0000 | 0.318 | 0.0105 |
| 8 | ATP5B | 0.563 | 0.0000 | 0.358 | 0.0037 |
| 9 | HADH | 0.562 | 0.0000 | 0.329 | 0.0078 |
| 10 | SUCLA2 | 0.542 | 0.0000 | 0.361 | 0.0034 |
| 11 | SDHB | 0.538 | 0.0000 | -0.046 | 0.7184 |
| 12 | DLD | 0.528 | 0.0000 | 0.327 | 0.0084 |
| 13 | CS | 0.523 | 0.0000 | 0.317 | 0.0106 |
| 14 | DLST | 0.510 | 0.0000 | 0.169 | 0.1806 |
| 15 | SDHA | 0.510 | 0.0000 | 0.428 | 0.0004 |
| 16 | SUCLG2 | 0.495 | 0.0000 | 0.328 | 0.0082 |
| 17 | DLAT | 0.493 | 0.0000 | 0.060 | 0.6354 |
| 18 | SUCLG1 | 0.465 | 0.0001 | 0.482 | 0.0001 |
| 19 | SLC25A3 | 0.461 | 0.0001 | 0.293 | 0.0188 |
| 20 | SLC25A11 | 0.457 | 0.0001 | 0.366 | 0.0029 |
| 21 | SLC25A4 | 0.456 | 0.0002 | 0.307 | 0.0137 |
| 22 | CYCS | 0.387 | 0.0016 | 0.328 | 0.0081 |
| 23 | OGDH | 0.383 | 0.0018 | 0.280 | 0.0248 |
| 24 | ACADM | 0.379 | 0.0020 | 0.472 | 0.0001 |
| 25 | COX5A | 0.353 | 0.0042 | 0.251 | 0.0453 |
| 26 | ACADS | 0.346 | 0.0051 | 0.405 | 0.0009 |
| 27 | CPT2 | 0.344 | 0.0054 | 0.552 | 0.0000 |
| 28 | ACADVL | 0.323 | 0.0092 | 0.516 | 0.0000 |
| 29 | NDUFS1 | 0.313 | 0.0119 | 0.184 | 0.1463 |
| 30 | ACADL | 0.312 | 0.0275 | 0.482 | 0.0004 |
| 31 | GPX4 | 0.309 | 0.0129 | -0.042 | 0.7398 |
| 32 | SLC25A5 | 0.298 | 0.0166 | 0.203 | 0.1080 |
| 33 | ETFDH | 0.282 | 0.0239 | 0.522 | 0.0000 |
| 34 | HADHA | 0.280 | 0.0250 | 0.620 | 0.0000 |
| 35 | IDH3A | 0.274 | 0.0285 | 0.311 | 0.0122 |
| 36 | ACAA2 | 0.239 | 0.0569 | 0.531 | 0.0000 |
| 37 | MTND5 | 0.226 | 0.0726 | -0.033 | 0.7960 |
| 38 | PDK1 | 0.176 | 0.1636 | 0.315 | 0.0113 |
| 39 | UCP2 | 0.163 | 0.1973 | 0.106 | 0.4042 |
| 40 | ETFB | 0.154 | 0.2234 | 0.402 | 0.0010 |
| 41 | SLC25A20 | 0.143 | 0.4352 | 0.143 | 0.4336 |
| 42 | UCP3 | 0.111 | 0.3844 | 0.509 | 0.0000 |
| 43 | ECI1 | 0.091 | 0.4736 | 0.143 | 0.2595 |
| 44 | HADHB | 0.063 | 0.6190 | 0.318 | 0.0105 |
| 45 | DECR1 | 0.059 | 0.6449 | 0.561 | 0.0000 |
| 46 | ECHS1 | 0.055 | 0.6647 | 0.202 | 0.1097 |
| 47 | SLC25A1 | 0.008 | 0.9470 | 0.188 | 0.1370 |
| 48 | PRDX6 | -0.016 | 0.8977 | -0.052 | 0.6839 |
| 49 | CPT1A | -0.072 | 0.5701 | 0.068 | 0.5945 |
| 50 | SLC25A10 | -0.080 | 0.5315 | 0.112 | 0.3796 |
| 51 | ACO2 | -0.106 | 0.4045 | 0.079 | 0.5340 |
| 52 | SLC25A22 | -0.118 | 0.3535 | 0.367 | 0.0028 |
| 53 | GSR | -0.127 | 0.3190 | 0.046 | 0.7162 |
| 54 | IDH2 | -0.178 | 0.1604 | 0.323 | 0.0092 |

**Table S3.** A list of proteins with a ρ_hierarchical_ ≥ 0 and p < 0.05 shared between different conditions as shown in Fig. 5E. LF (-)RW, low-fat without running wheel; LF (+)RW, low-fat with running wheel; HFS (-)RW, high-fat sucrose without running wheel; HFS (+)RW, high-fat sucrose with running wheel.

| All | LF (-)RW, HFS(-)RW, HFS(+)RW | LF (-)RW,  HFS(-)RW,  LF (+)RW | LF (-)RW, LF (+)RW, HFS(+)RW | LF (-)RW, HFS(-)RW | LF (-)RW, HFS (+)RW | HFS (-)RW, HFS (+)RW | HFS (+)RW |
| --- | --- | --- | --- | --- | --- | --- | --- |
| Atp5b | Cox5a | Dlat | Fh | Mtnd5 | Slc25a5 | Cycs | Idh3a |
| Cs | Dld | Dlst | Suclg2 |  | Sucla2 | Mdh2 | Ndufs1 |
| Gpx4 | Pdha1 | Sdhb |  |  |  | Ogdh | Slc25a1 |
| Slc25a3 | Slc25a4 |  |  |  |  | Sdha |  |
| Uqcrc2 | Slc25a11 |  |  |  |  | Suclg1 |  |
|  | Sod2 |  |  |  |  |  |  |

**Figure S1 MHC content**


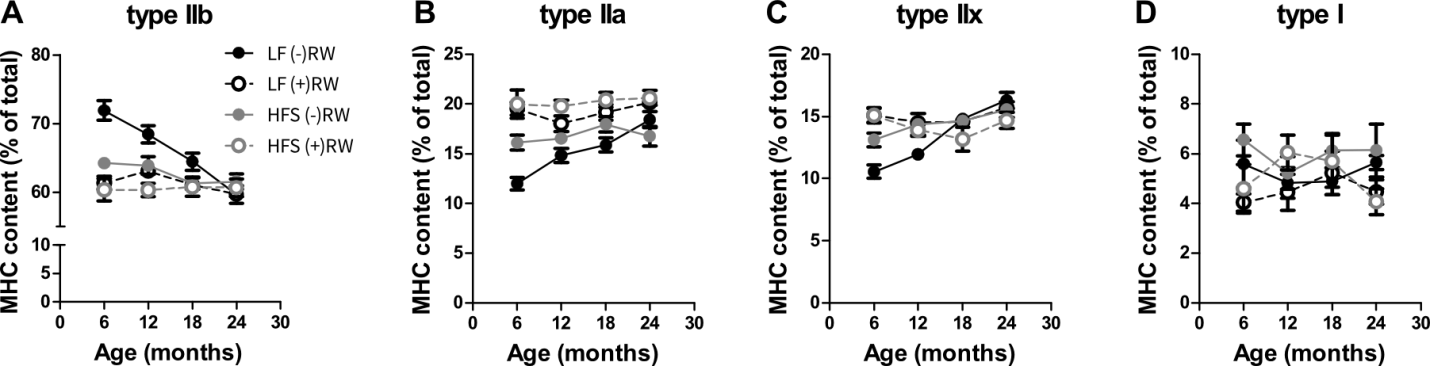


**Figure S1 Myosin heavy chain (MHC) composition of quadriceps muscle.** Linear regression analysis showed that the fractional contribution of fast-twitch glycolytic type IIb MHC isoform to the total MHC pool decreased in response to high-fat sucrose (HFS) diet (p < 0.01, β = -0.238), voluntary running wheel (RW) activity (p < 0.001, β = -0.422), and age (p < 0.001, β = -0.368). This decrease was largely compensated by an increase in the fast-twitch oxidative-glycolytic type IIa MHC isoform in response to HFS diet (p < 0.01, β = 0.207), voluntary RW activity (p < 0.001, β = 0.609), and age (p < 0.001, β = 0.267), and oxidative-glycolytic type IIx MHC isoform in response to RW activity (p < 0.05, β = 0.178) and age (p < 0.001, β = 0.384), but not HFS diet (p = 0.798, β = 0.022). The content of slow-twitch oxidative type I MHC was only significantly negatively affected by age (p < 0.05, β = -0.002) without significant effect by HFS diet and RW activity. Data are represented as average of n = 7-8 ± SEM. LF (-)RW, low-fat without running wheel; LF (+)RW, low-fat with running wheel; HFS (-)RW, high-fat sucrose without running wheel; HFS (+)RW, high-fat sucrose with running wheel.

**Figure S2
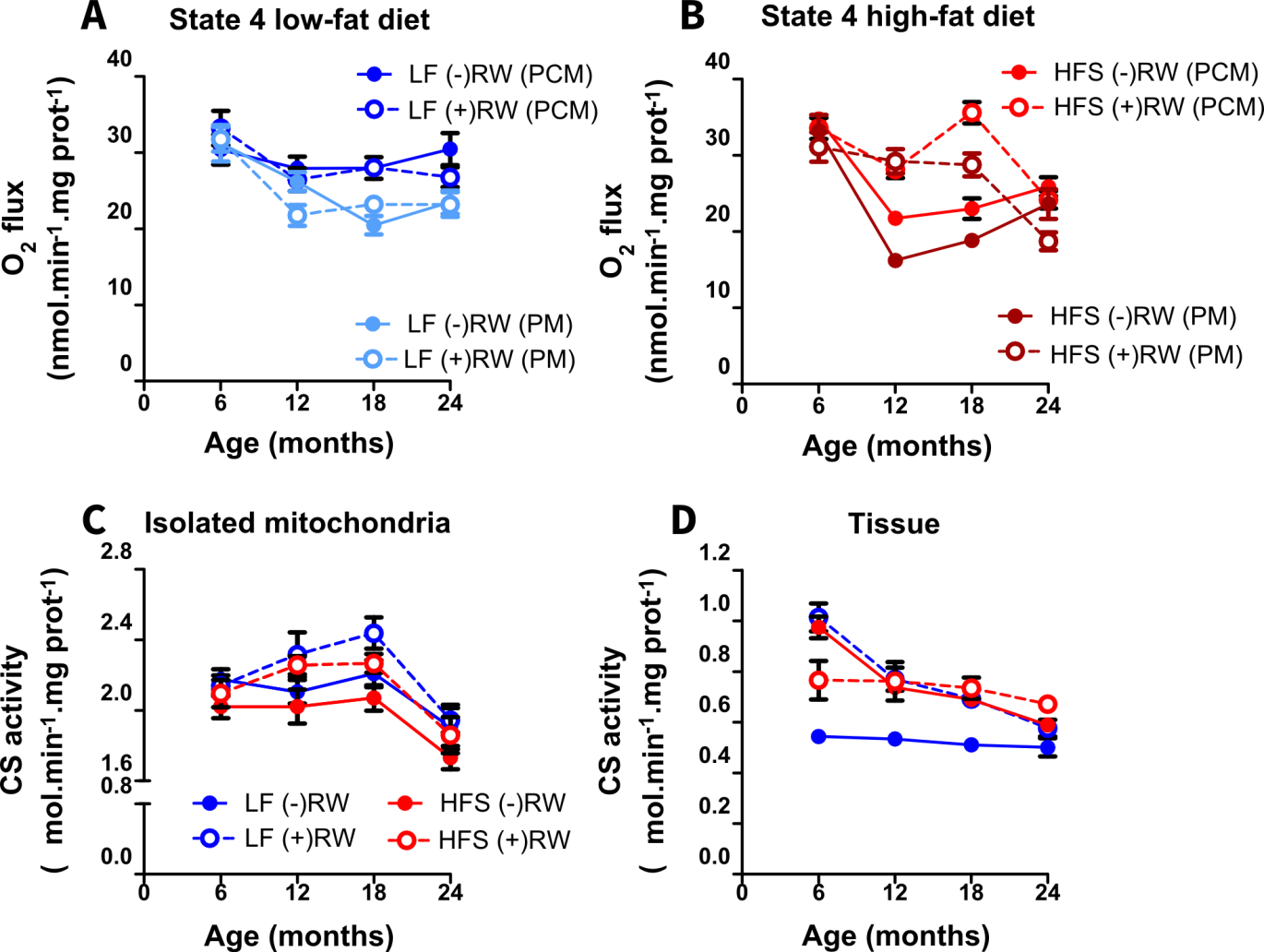
**

**Figure S2. The effect of high-fat sucrose (HFS) diet, running wheel (RW) activity, and age on the respiratory function of skeletal muscle mitochondria.** Basal O_2_ flux (state 4) in isolated skeletal muscle mitochondria from (**A**) low-fat (LF) and (**B**) high-fat/sucrose (HFS) diet-fed animals. Data are average of n = 7-8 per group ± SEM. Linear regression analysis for pyruvate plus malate (PM) data: p(HFS) = 0.512, β(HFS) = -0.051; p(RW) = 0.040, β(RW) = 0.160; p(Age) < 0.001, β(Age) = -0.476. Linear regression for analysis for palmitoyl-CoA plus L-carnitine plus malate (PCM) data: p(HFS) = 0.387, β(HFS) = -0.072; p(RW) = 0.056, β(RW) = 0.160; p(Age) < 0.001, β(Age) = -0.318.

Citrate synthase (CS) activity in (**C**) isolated skeletal muscle mitochondria and (**D**) total skeletal muscle homogenate. Data are average of n = 7-8 per group ± SEM. Linear regression analysis for CS activity in isolated mitochondria: p(HFS) = 0.011, β(HFS) = -0.210; p(RW) = 0.005, β(RW) = 0.234; p(Age) = 0.002, β(Age) = -0.262. Linear regression analysis for CS activity in muscle tissue homogenate: p(HFS) < 0.001, β(HFS) = 0.326; p(RW) < 0.001, β(RW) = 0.358; p(Age) < 0.001, β(Age) = -0.483. LF (-)RW, low-fat control; LF (+)RW, low-fat running wheel; HFS (-)RW, high-fat/sucrose control; HFS (+)RW, high-fat/sucrose running wheel.

**Figure S3**


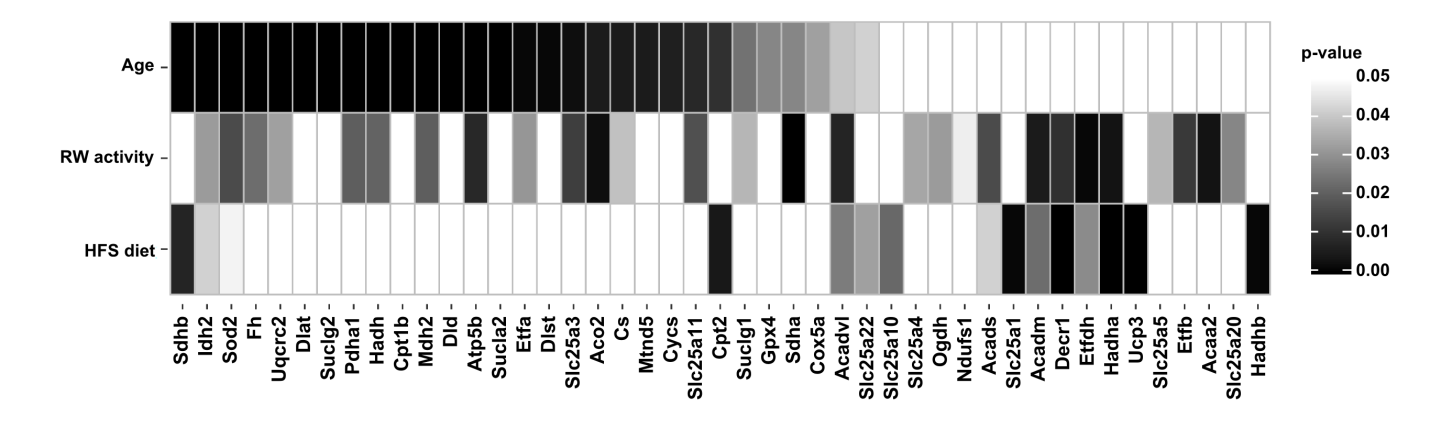


**Figure S3. Multiple linear regression of targeted proteomics.** Proteins that are significantly (p-value < 0.05) affected by at least one of the three factors (age, running wheel (RW) activity or high-fat sucrose (HFS) diet) are shown. Proteins with white background are not significantly changed for that factor.

**Figure S4**


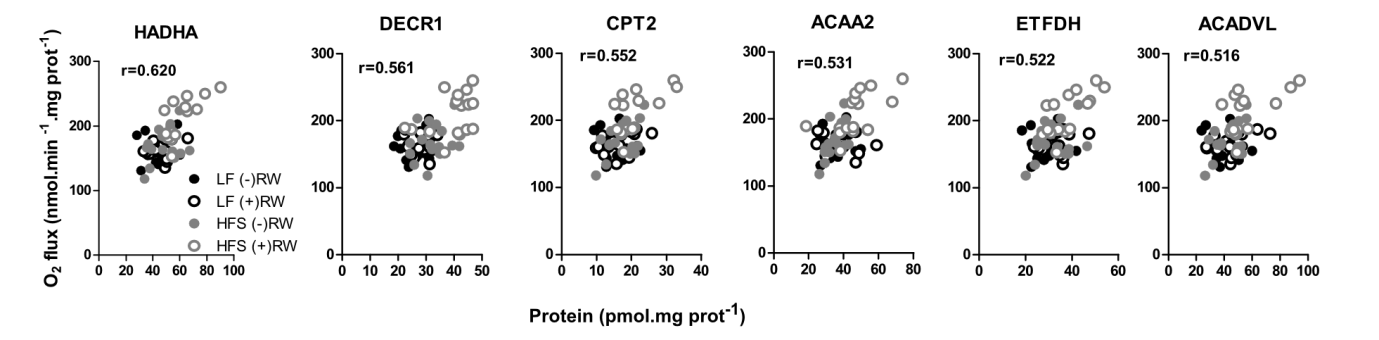


**Figure S4. Correlation between mitochondrial protein concentration and mitochondrial O_2_ flux driven by palmitoyl-CoA plus L-carnitine plus malate (PCM).** Six proteins meaningful for PCM oxidation with the highest Pearson correlation coefficient are shown. Data are n = 4 mice per experimental group and time point.

**Figure S5**


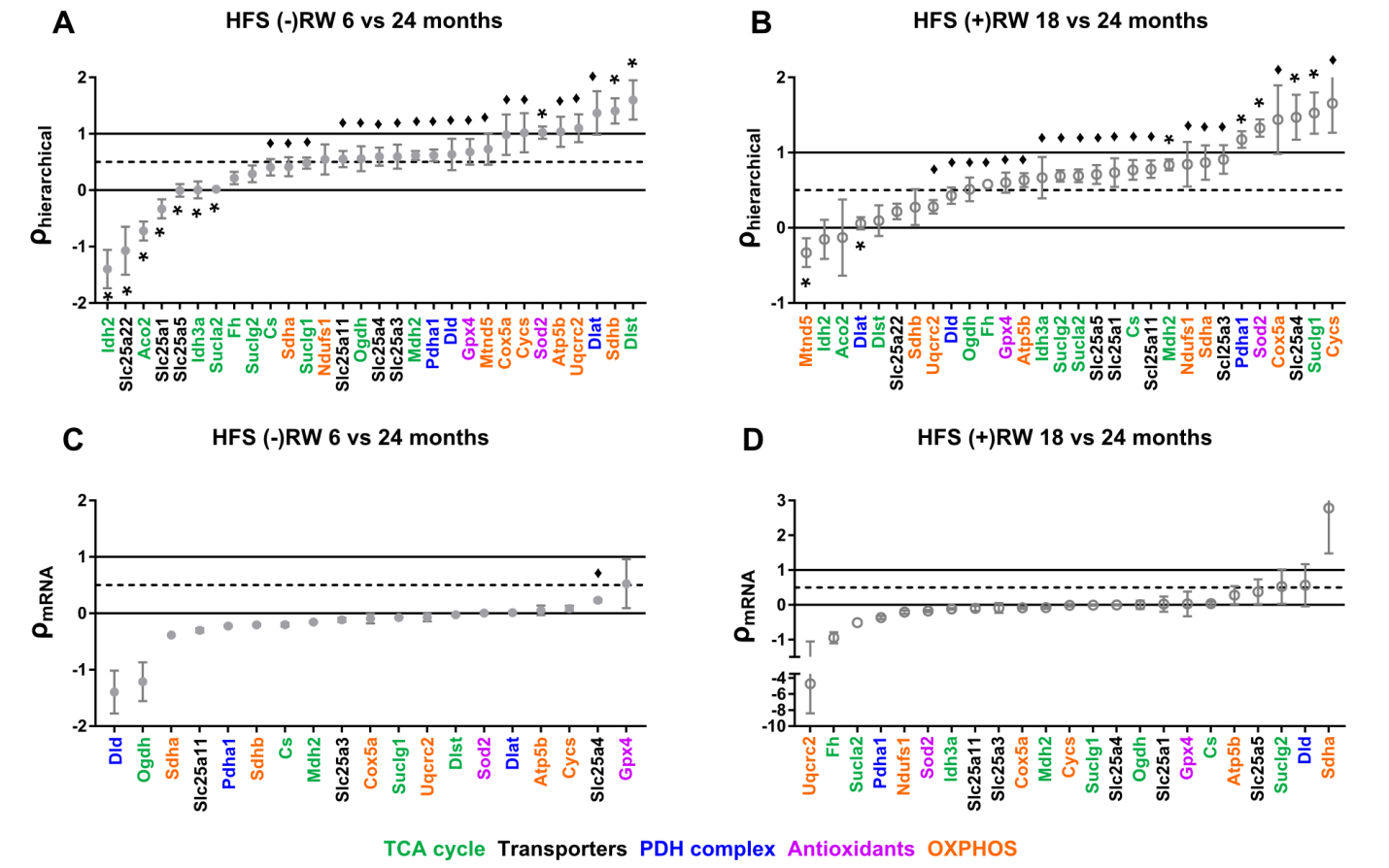


**Figure S5. The regulation of O_2_ flux in skeletal muscle mitochondria of ageing mice. (A, B)** Hierarchical regulation coefficient *ρ_h_* for HFS (-)RW (A) and HFS (+)RW mice (B). A coefficient of 1 means that the change of flux during ageing can be explained completely by the change in protein concentration, whereas a coefficient of 0 means that flux is completely metabolically regulated (n = 4 per group). **(C, D)** Transcriptional regulation coefficient *ρ_mRNA_* for HFS (-)RW (C) and HFS (+)RW (D) mice. Only proteins with a ***ρ_h_* > 0 and p < 0.05** were taken into account. The coefficients are based on n = 3 for mRNA, n = 4 for protein. The average ± SD is plotted in ascending order independently for each condition for all regulation coefficients. * (*ρ_h_* ≠ 0.5), ♦ (*ρ_h_* > 0) each with adjusted p value < 0.05. **If the same protein was significant for **ρ_h_* ≠ 0.5 and ♦ *ρ_h_* > 0, then only * was indicated.** The enzymes that belong to the same metabolic pathway are highlighted in the same colour as pathways represented in Fig. 1. HFS (-)RW, high-fat/sucrose control; HFS (+)RW, high-fat/sucrose running wheel.

**Figure S6**

**
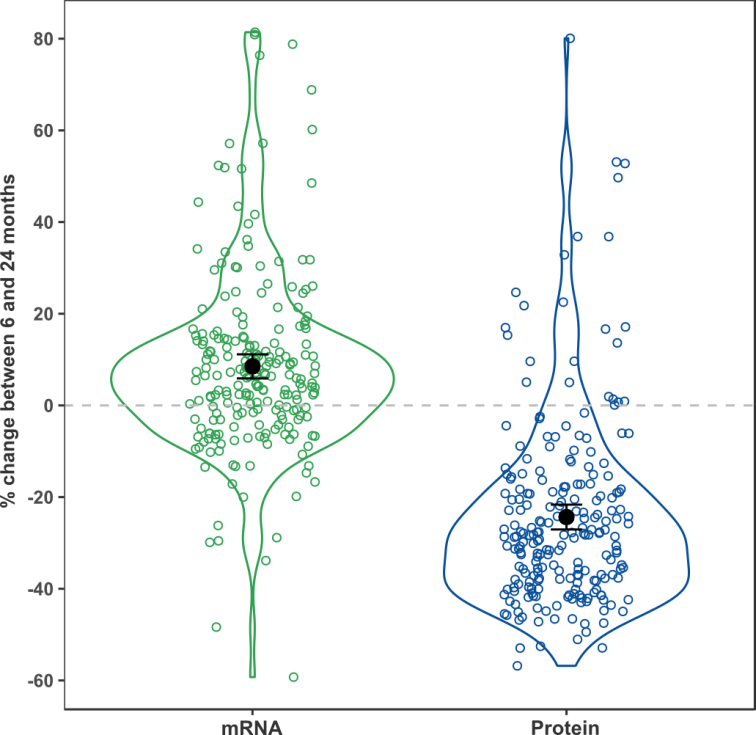
**

**Figure S6. Uncoupling of protein and mRNA expression with age.** Percentage of change between 6 and 24 months in either mRNA abundance (left) or protein concentration (right) for all four conditions. The black dot indicates the mean change in percentage and the error bars show the 95% confidence interval. Only mRNA coding for measured proteins were taken into account.

**Figure S7.**


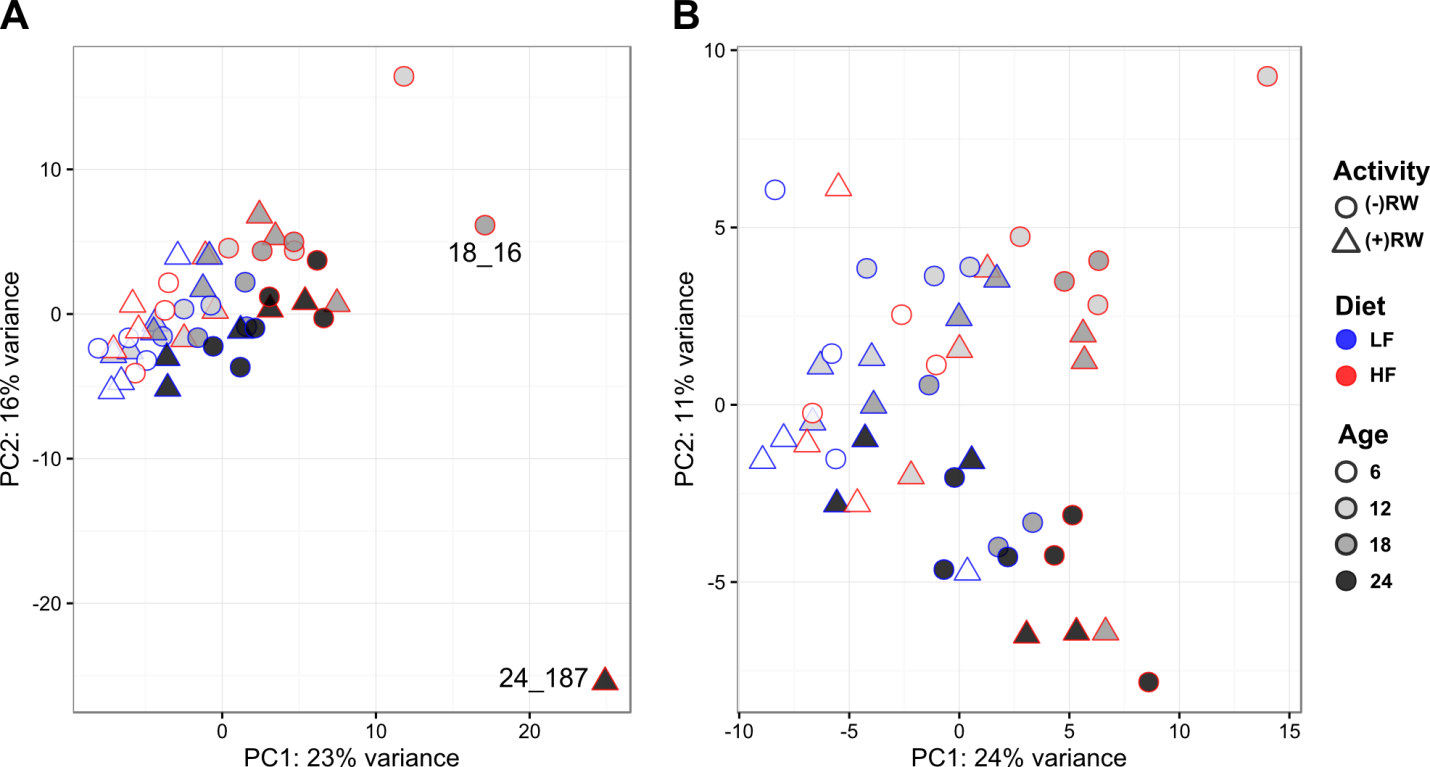


**Figure S7:** **PCA of muscle mRNA expression. A)** After consulting additional body weight and running wheel data mice 18_16 and 24_187 were identified as outliers. **B)** PCA plot after removal of identified outliers. The low-fat diet (LF) group is shown in blue and high-fat sucrose diet (HFS) group in red; (-)RW and circle indicate a group without access to running wheel, the (+)RW and triangle indicates a group with access to a running wheel. The age is indicated with a grayscale ranging from white at 6 months to black at 24 months of age.

**
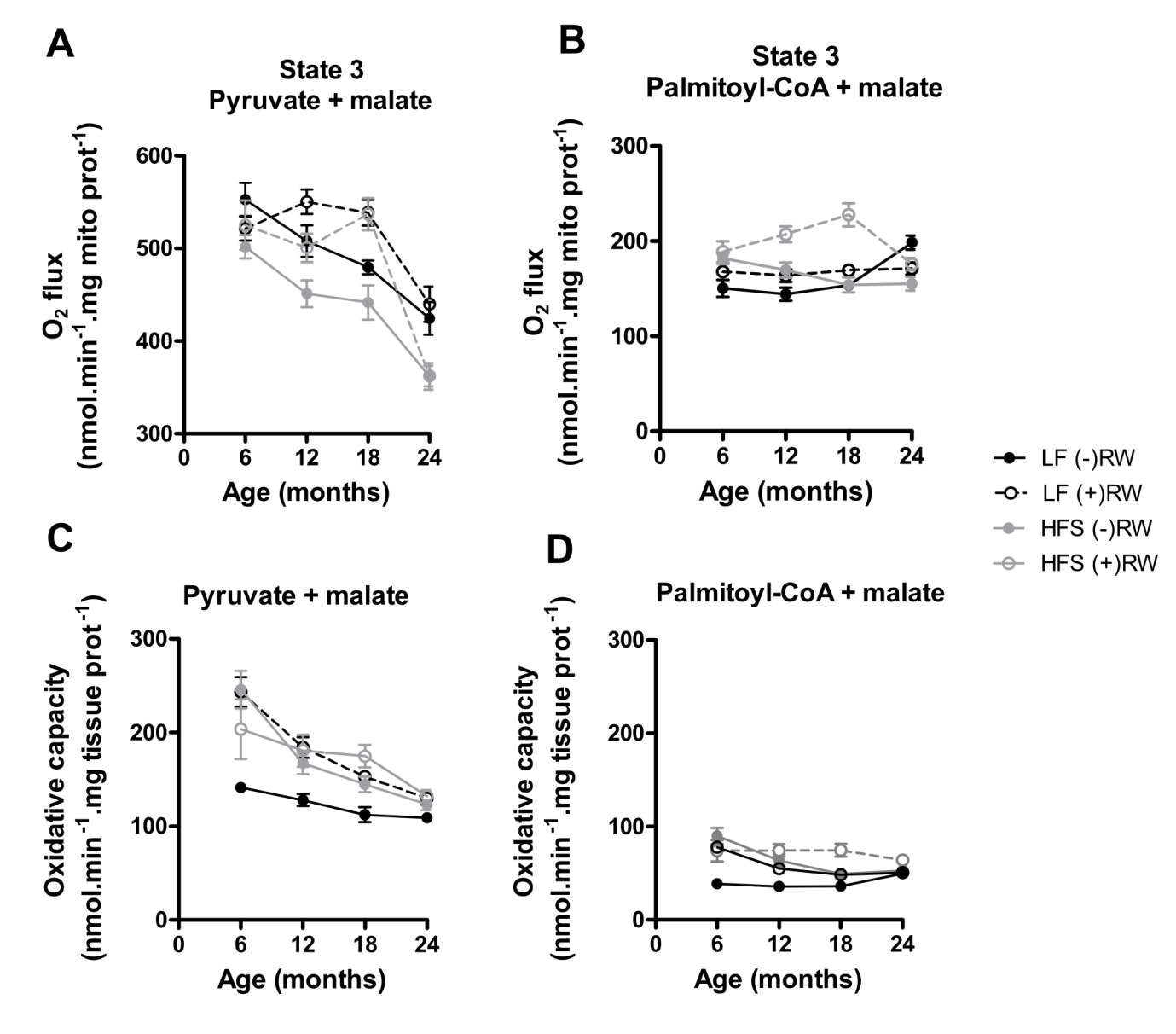
**

**Figure S8: Skeletal muscle mitochondrial properties. Same data as Fig 3, but here shown per substrate for easier comparison** (**A, B**) Maximal ADP-stimulated O_2_ flux (state 3) in isolated skeletal muscle mitochondria oxidizing (A) pyruvate plus malate or (B) palmitoyl-CoA plus L-carnitine plus malate in low-fat and high-fat sucrose diet groups. **(C, D)** Maximum skeletal muscle oxygen flux capacity expressed per total tissue protein, determined as the state 3 O_2_ flux in isolated mitochondria multiplied by the mitochondrial protein content in skeletal muscle. Data are averages of n = 7-8 mice per experimental group and time point ± SEM. Experimental group abbreviations as in Fig. 2.

**Figure S9**

**
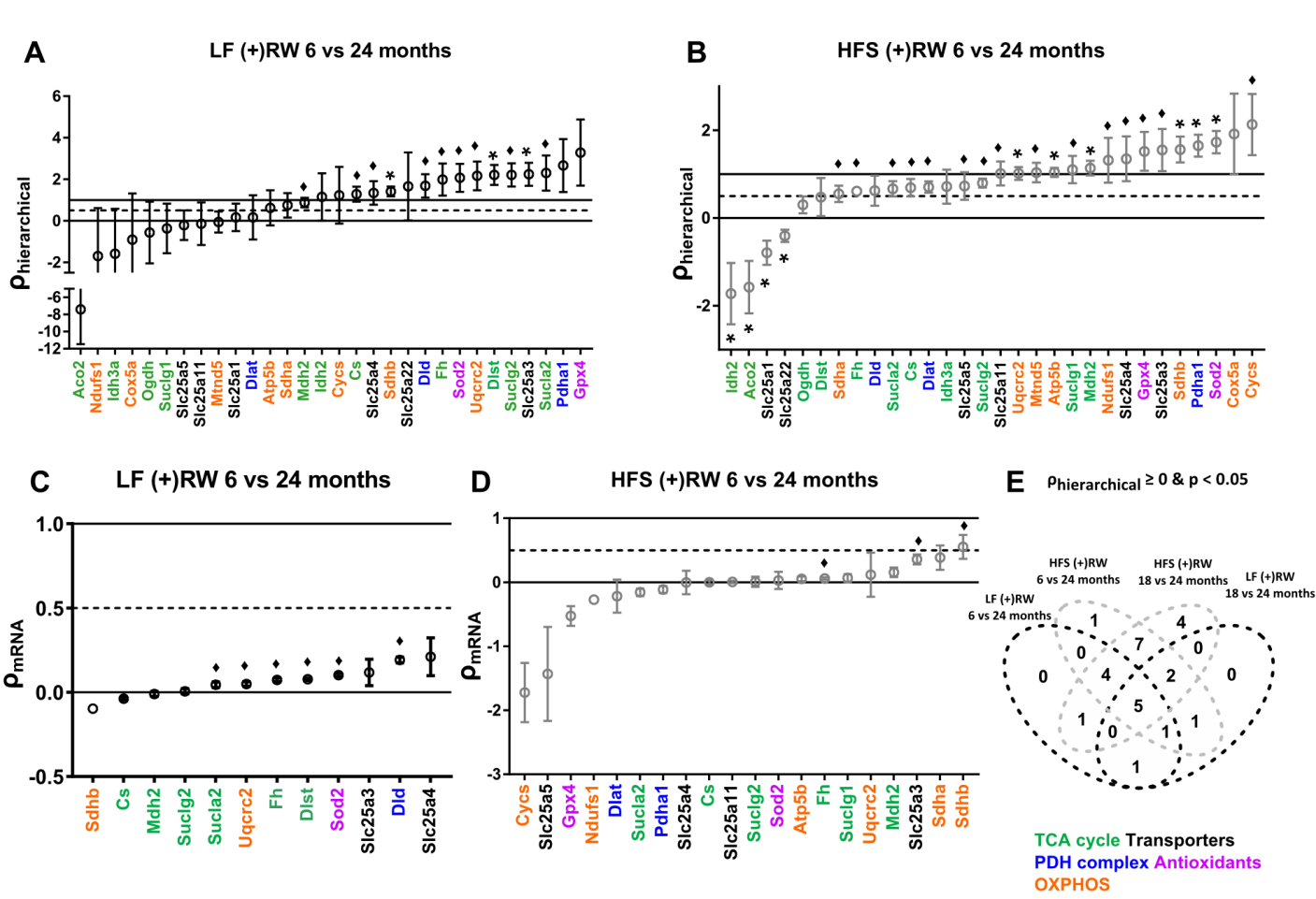
**

**Figure S9. The regulation of O_2_ flux in skeletal muscle mitochondria of ageing mice with RW. (A, B)** Hierarchical regulation coefficient *ρ_h_* for LF (+)RW (A) and HFS (+)RW mice (B) (n = 4 per group). **(C, D)** Transcriptional regulation coefficient *ρ_mRNA_* for LF (+)RW (C) and HFS (+)RW (D) mice. Only proteins with a ***ρ_h_* > 0 and p < 0.05** were taken into account in **C, D**. The *ρ_mRNA_* values are based on n = 3 for mRNA, n = 4 for protein. The average ± SD is plotted in ascending order independently for each condition for all regulation coefficients. * (*ρ_h_* ≠ 0.5), ♦ (*ρ_h_* > 0) each with adjusted p value < 0.05. If the same protein was significant for **ρ_h_* ≠ 0.5 and ♦*ρ_h_* > 0, then only * was indicated. The enzymes that belong to the same metabolic pathway are highlighted in the same colour as pathways represented in Fig. 1. **(E)** Venn diagram showing proteins with a *ρ_h_* > 0 and p < 0.05 for all (+)RW comparisons. **Proteins that are shared between all groups are CS, FH, SLC25A3, SUCLG2 and UQCRC2. The large degree of overlap between the 6 vs 24 months and 18 vs 24 months comparisons shows that the choice for 6 or 18 months as the reference time point had only minor effects (7 reactions overlapping between 6 vs 24 months and 18 vs 24 months in the LF(+)RW groups, in which 12 and 10 reactions had a hierarchical component, respectively; and even 18 overlapping between 6 vs 24 months and 18 vs 24 months in the HFS(+)RW groups, in which 21 and 23 reactions had a hierarchical component). Of the proteins with a hierarchical regulation in three or four groups as discussed in the Discussion section, most also had a significant hierarchical regulation in the 6 vs 24 months comparison (cf. Fig S9A-B to Fig 5B and Fig S5B).** LF (+)RW, low-fat running wheel, HFS (+)RW, high-fat/sucrose running wheel.
