# Supplementary material for: Running‐wheel activity delays mitochondrial respiratory flux decline in aging mouse muscle via a post‐transcriptional mechanism
Source: Aging Cell. 2017 Nov 9;17(1):e12700. doi: 10.1111/acel.12700 (PMC5770778; doi:10.1111/acel.12700)
Supplement: Supplementary file 13 [file ACEL-17-na-s013.docx]

**Supplemental Experimental procedures**

*Running wheel intervention*

The cages of LF (+)RW and HFS (+)RW mice contained an in-house made plastic running wheel (diameter 13.3 cm) equipped with a small magnet and a magnetic relay attached to the cage and connected to the PC. The running wheel activity was sampled continuously and stored in one minute bins by a Circadian Activity Monitor System (CAMS, by H.M. Cooper and J.A. Cooper, INSERM U846, Department of Chronobiology, Bon, France). The raw data was imported into a custom made excel macro package (ACTOVIEW, C.K. Mulder, Department of Molecular Neurobiology, Groningen, Netherlands) and monthly averages (m.h^-1^) were calculated.

*Skeletal muscle triglyceride content*

Quadriceps muscles were powdered in liquid nitrogen and 10% (w/v) homogenates in PBS (pH 7.4) were prepared. Total tissue lipids were extracted according to (Bligh & Dyer 1959). Triglyceride content was determined using a commercial triglyceride kit (Roche Diagnostics, Mannheim, Germany) according to the manufacturer’s recommendations and expressed in μmol/g wet muscle weight.

*Skeletal muscle myosin heavy chain (MHC) composition*

Quadriceps muscle homogenates (10%, w/v) were prepared in a buffer containing 250 mM sucrose, 100 mM KCl, 5 mM EDTA, 20 mM Tris, and a protease inhibitor cocktail (1:200; Sigma-Aldrich, Zwijndrecht, The Netherlands), at pH 6.8. MHC isoforms in the homogenates were resolved using SDS-PAGE and quantified as described in (Talmadge & Roy 1993).

*Isolation of mitochondria and high-resolution respirometry*

Skeletal muscle mitochondria were isolated by differential centrifugation as described elsewhere (van den Broek et al. 2010). The protein content was determined using BCA protein assay kit (Pierce, Thermo Fisher Scientific Inc., Rockford, IL, USA). The O_2_ fluxes in isolated mitochondria were measured at 37 °C in a two-channel high-resolution Oroboros oxygraph-2 k (Oroboros, Innsbruck, Austria). The oxidizable substrates were: (i) 5 mM pyruvate plus 2 mM malate, or (ii) 25 μM palmitoyl-CoA plus 2 mM L-carnitine plus 2 mM malate. The O_2_ fluxes were expressed in nmol/min/mg mitochondrial protein.

*Citrate synthase activity and mitochondrial content*

Citrate synthase activity was determined in isolated mitochondria and quadriceps muscle homogenates (10%, w/v) in PBS (pH 7.4). The samples were sonicated for 30 s in the pulse mode (pulse duration 1 s, interval between the pulses 1 s, amplitude 20%) on ice, followed by 10 min centrifugation at 1000 *g*, 4 °C. Citrate synthase activity in the supernatant was determined spectrophotometrically by measuring the formation of 5-thio-2-nitrobenzoic acid at 412 nm at 37 °C (Srere et al. 1963). The assay mixture contained 0.1 M Tris (pH 8.1), 5 mM triethanolamine-HCl, 0.05 mM EDTA, 0.1% triton-X100, 0.5 mM oxaloacetate, 0.1 mM dithionitrobenzoic acid. The reaction was started with 0.5 mM acetyl-CoA. Enzyme activities were expressed in μmol/min/mg mitochondrial or total tissue protein. Mitochondrial protein content per cellular protein was calculated as a ratio of citrate synthase (CS) activity in the total tissue to the CS activity in mitochondrial fraction, both expressed per protein in the respective fractions. This should be considered an approximation, since it depends on the purity of the mitochondrial preparation. For the conversion of fluxes or concentrations normalized per mitochondrial protein to whole tissue, the purity of the preparation does not matter, however, since the enrichment factor for CS is the same as for other mitochondrial proteins.

*Mitochondrial DNA (mtDNA) copy number*

Total DNA was isolated from 10-20 mg of tissue with the GenElute Mammalian Genomic DNA Miniprep Kit (Sigma-Aldrich, Zwijndrecht, The Netherlands). Relative mtDNA copy number was determined by assessing the copy number of mitochondrial genome-encoded 16S ribosomal RNA gene (*mt-Rnr2*) relative to a single copy nuclear glyceraldehyde-3-phosphate dehydrogenase gene (*Gapdh*) on the 7900HT Fast Real-Time PCR system (Applied Biosystems, Waltham, Massachusetts, MA, USA) as described in (Reijne et al. 2016).

*Composition of assay medium for high-resolution respirometry*

The assay medium (MiR05) contained 110 mM sucrose, 60 mM potassium lactobionate, 20 mM taurine, 20 mM HEPES, 0.5 mM EGTA, 10 mM KH_2_PO_4_, 3 mM MgCl_2_, 1 mg/ml bovine serum albumin, at pH 7.1. The oxidizable substrates were: (i) 5 mM pyruvate plus 2 mM malate, or (ii) 25 μM palmitoyl-CoA plus 2 mM L-carnitine plus 2 mM malate. Maximal ADP-stimulated O_2_ flux (state 3) was achieved by adding 1.5 U/ml hexokinase, 12.5 mM glucose and 1 mM ATP. The basal O_2_ flux (state 4) was determined after blocking ADP phosphorylation with 1.25 μM carboxyatractyloside. Data acquisition and analysis were performed in DatLab software version 5.1 (Oroboros, Innsbruck, Austria).

*Targeted quantitative mitochondrial proteomics*

A set of 54 mitochondrial proteins involved in substrate transport, oxidative phosphorylation (OXPHOS), fatty acid β-oxidation, tricarboxylic acid cycle, and antioxidant activity were quantified in isolated mitochondria using isotopically labelled standards (^13^C-labeled lysines and arginines) derived from synthetic protein concatemers (QconCAT) (PolyQuant GmbH, Bad Abbach, Germany) as described by Wolters et. al. (Wolters et al. 2016). In brief, mitochondrial samples (50 μg protein) were mixed with 1.5 ng of QconCAT per 1 μg of total mitochondrial protein. After reduction with 10 mM dithiothreitol and alkylation with 55 mM iodoacetamide the protein extract was subjected to in-gel tryptic digestion (1:100 g/g). The peptides listed in (Wolters et al. 2016) were targeted and analyzed by a triple quadrupole mass spectrometer (MS) equipped with a nano-electrospray ion source (TSQ Vantage, Thermo Scientific). The chromatographic separation (gradient 100 min) of the peptides was performed by liquid chromatography on a nano-UHPLC system (Ultimate UHPLC focused, Dionex). The MS traces were manually curated in the Skyline software (MacLean et al. 2010) prior to integration of the peak areas for quantification. The sum of all transition peak areas for the endogenous peptides and isotopically labeled QconCAT-peptide standards was used to calculate the ratio between the endogenous and standard peptides. The concentrations of the endogenous peptides were calculated from the known concentrations of the standards and expressed in pmol/mg of mitochondrial protein.

*Statistical analysis*

The data up to now are presented as means ± SEM. The normality of data was assessed by a Shapiro-Wilk test. If the data were not normally distributed, they were log-transformed before further analyses. The general effects of age, HFS feeding and RW activity were determined using linear regression analysis of complete time-courses. For this analysis, the p value and standardized coefficient β (the sign of which indicates the direction of the change) are shown for each protein and effector. The significance of the differences between the sedentary and running wheel groups - at individual time point for a particular diet - was assessed by a student’s t-test for unpaired data. All analyses were performed with IBM SPSS Statistics version 23.0 (SPSS Inc., Chicago, IL, USA) unless otherwise stated. The level of significance was set at p < 0.05.

*RNA isolation and RNA-Sequencing*

Total RNA was extracted from 30-55 mg of quadriceps muscle (n = 3 per experimental group) using Trizol reagent (Invitrogen, Carlsbad, CA, USA). The purity and concentration of RNA was determined using NanoDrop ND-1000 (ISOGEN Life Science, De Meern, The Netherlands), followed by the assessment of RNA integrity and concentration by capillary electrophoresis using PerkinElmer LabChipGX (PerkinElmer, Waltham, MA, USA). Samples were considered for RNA sequencing when the integrity score was above 7, the 5S peak was small and both the 18S and 28S peaks were clearly distinguishable. Sequence libraries were generated using the TruSeq RNA sample preparation kits (Illumina, San Diego, CA, USA) using the Sciclone NGS Liquid Handler (PerkinElmer, Waltham, MA, USA). In case of contamination of adapter duplexes an extra purification of the libraries was performed with the automated agarose gel separation system Labchip XT (PerkinElmer, Waltham, MA, USA). The obtained cDNA fragment libraries were sequenced on an Illumina HiSeq2500 using default parameters (single read 1x50bp, High Output modus) in pools of eight samples per lane.

*Analysis of RNA-sequencing data*

Sequencing reads were mapped to the mouse genome (mm10 assembly) using STAR (version 2.3.0) (Dobin et al. 2013). The aligner was provided with a file containing junctions from Ensembl GRCm38.74. In total, there were 1 billion reads from 72 samples. Htseq-count of the Python package HTSeq (version 0.5.4p3) was used to quantify the read counts per gene based on annotation version GRCm38.74, using the default union-counting mode (The HTSeq package, http://www-huber.embl.de/users/anders/HTSeq/doc/ overview.html).

We performed PCA analysis to check for batch effects and screen for outlier samples. We used the PCA function from the DESeq2 package (version 1.10.1) in R (version 3.2.5) on rlog transformed raw counts. When a sample explained a large part of the variance, we consulted the bodyweight and running wheel data, to determine if the sample should be considered an outlier. RPKM normalized read counts corresponding to the 54 mitochondrial proteins were used for regulation analysis.

*Background on Regulation Analysis*

The rate of an enzyme reaction is regulated by multiple factors upon environmental perturbations. Regulation analysis (Kuile & Westerhoff 2001; Daran-Lapujade et al. 2007) estimates to what extent each factor contributes to the change in enzyme rate. The regulation upon a transition from one steady state to another can be dissected into two main contributions: hierarchical regulation and metabolic regulation. Hierarchical regulation comprises the entire gene-expression cascade all the way from DNA, via mRNA, to the final enzyme concentration. Metabolic regulation is a combination of all remaining metabolic effects including concentrations of substrates, products, and allosteric effectors, as well as the corresponding affinity constants.

Mathematically, the combined effect of the metabolic and hierarchical regulation on the enzyme rate *v* is described by:

$$v=e\cdot g(\boldsymbol{M}, \boldsymbol{K})$$

in which *e* is the enzyme concentration and *g(****M, K****)* is a function of the vector of all metabolite concentrations ***M*** acting on the enzyme and the vector of kinetic constants ***K.*** To obtain the relative contribution of hierarchical (gene-expression) and metabolic regulation, we performed a logarithmic transformation, calculated the difference (Δ) between the steady-state rates before and after a specific perturbation, and finally divided each term in the equation by the logarithmic change in enzyme rate, resulting in (Kuile & Westerhoff 2001):

$$1= \frac{\Delta lne}{\Delta ln(v)}+ \frac{\Delta ln(g\left( \boldsymbol{M}, \boldsymbol{K} \right))}{\Delta ln(v)}= \rho_{h}+ \rho_{m}$$

The first term represents the hierarchical regulation coefficient *ρ_h_* and the second term the metabolic regulation coefficient *ρ_m_*. Together they explain the change in enzyme rate completely.

For each enzyme *i*, we calculated the hierarchical regulation coefficients (*ρ_h, i_*) as follows:

$$\rho_{h,i}= \frac{{lne}_{i,age1}-{lne}_{i,age2}}{\ln v_{i,age1}-\ln v_{i,age2}}$$

where *e* is the enzyme concentration and *v* is the flux (both expressed per mitochondrial protein). We subtracted samples of 24 months old mice (*age2*) from samples of younger mice (*age1*); for (‑)RW condition we used the 6 months’ time point, for (+)RW condition the 18 months’ time point. We assumed that all enzyme rates *v* were proportional to the measured oxygen flux. For different time points (*n* = 4 each) measurements were done in different mice, since the mice were sacrificed at the indicated ages. As all mice were independent, we calculated all combinations of ratios for the different age combinations. This resulted in 16 values for each *ρ_h,i_*. To estimate the mean ρ_h,i_ we performed bootstrapping by randomly resampling the 16 ratios 1000 times with replacement. The advantage of bootstrapping is that it does not assume any underlying distribution (Efron & Tibshirani 1993). Then the standard deviation of the estimated mean values was calculated. For each protein we calculated if it had a significantly higher or lower *ρ_h_* value than 0.5 using a two-tailed t-test. Similarly, we calculated if a protein had a *ρ_h_* > 0 or *ρ_h_* < 1 using a one-sided t-test, to test for shared regulation. Afterwards, we corrected for multiple testing using the Benjamini-Hochberg procedure. The level of significance was set at adjusted p < 0.05.

**If the average *ρ_h_* was above 0 with p < 0.05**, we zoomed further into the hierarchical regulation. Assuming a pseudo-steady state, the hierarchical regulation coefficient can be further dissected into its individual processes (transcription, translation and enzyme stability). Since we only measured mRNA and enzyme concentrations, we simplified this analysis as follows. As in the original derivation (Daran-Lapujade et al. 2007) we assumed the rate of enzyme synthesis to be proportional to the mRNA concentration with rate constant *k_translation_* and the rate of enzyme degradation proportional to the enzyme concentration *e* with rate constant *k_degradation_*. At steady state the rate of synthesis equals that of degradation:

$$k_{translation}\cdot\left[ ribosome \right]\cdot[mRNA]=k_{degradation}\cdot e$$

Solving the equation for *e*, again performing a logarithmic transformation, taking the difference between the steady states, and now dividing by *e*, results in (Daran-Lapujade et al. 2007):

$$1= \underset{\rho_{translation}}{\underbrace{\frac{\Delta lnk_{translation}}{\Delta\ln e}+\frac{\Delta ln[ribosome]}{\Delta\ln e}}}-\underset{\rho_{degradation}}{\underbrace{\frac{\Delta\ln k_{degradation}}{\Delta\ln e}}}+\underset{\rho_{mRNA}}{\underbrace{\frac{\Delta\ln\left[ mRNA \right]}{\Delta\ln e}}}$$

Based on the available proteome and transcriptome data *ρ_mRNA_* was calculated and it follows from the equation that the contribution of protein degradation and synthesis to the overall protein concentration equals 1 minus *ρ_mRNA_*.

The transcriptional regulation coefficients (ρ_mRNA,i_) were calculated as follows:

$$\rho_{mRNA,i}= \frac{{\ln\left[ mRNA \right]}_{i, age1}- {\ln\left[ mRNA \right]}_{i, age2}}{{\ln\left[ e_{tissue} \right]}_{i, age1}- {\ln\left[ e_{tissue} \right]}_{i, age2}}$$

For that we used the proteomics data mentioned above and the mRNA abundance measured by RNASeq. Since the mRNA abundance was measured at the tissue level, we transformed the protein concentrations also to the tissue level by multiplying the abundance of the proteins in the mitochondrial fraction with the mitochondrial protein content relative to total cellular protein content. The latter was calculated as the ratio of CS activity in the total tissue divided by that in the mitochondrial fractions, both expressed per protein in the respective fractions. In this case, not only the samples for different ages were from different mice, but also mRNA abundance (*n* = 3) was measured in different mice than protein concentrations (*n* = 4) . Therefore, we calculated all possible combinations of the protein and mRNA concentrations at the two different ages, resulting in 144 ρ_mRNA,i_ ratios per protein. The mean ρ_mRNA,i_ and standard deviation were calculated as described above by bootstrapping with replacement. The significant ρ_mRNA,i_ were calculated as described above using a t-test. We calculate the ρ_mRNA,i_ for all proteins **with mean ρ_h_ significantly above 0**, except for NADH-ubiquinone oxidoreductase chain 5 (mt-nd5). Results for mt-nd5 mRNA were discarded during the alignment process due to duplicate mapping. All calculations were performed in R (version 3.2.5) (R Core Team, 2016).

Bligh EG & Dyer WJ (1959) A rapid method of total lipid extraction and purification. *Can. J. Biochem. Physiol.* 37, 911–7.

Daran-Lapujade P, Rossell S, van Gulik WM, Luttik M a H, de Groot MJL, Slijper M, Heck AJR, Daran J-M, de Winde JH, Westerhoff H V, Pronk JT & Bakker BM (2007) The fluxes through glycolytic enzymes in Saccharomyces cerevisiae are predominantly regulated at posttranscriptional levels. *Proc. Natl. Acad. Sci. U. S. A.* 104, 15753–8.

Dobin A, Davis C a, Schlesinger F, Drenkow J, Zaleski C, Jha S, Batut P, Chaisson M & Gingeras TR (2013) STAR: ultrafast universal RNA-seq aligner. *Bioinformatics* 29, 15–21.

Kuile BH & Westerhoff H V. (2001) Transcriptome meets metabolome: Hierarchical and metabolic regulation of the glycolytic pathway. *FEBS Lett.* 500, 169–171.

MacLean B, Tomazela DM, Shulman N, Chambers M, Finney GL, Frewen B, Kern R, Tabb DL, Liebler DC & MacCoss MJ (2010) Skyline: An open source document editor for creating and analyzing targeted proteomics experiments. *Bioinformatics* 26, 966–968.

Reijne AC, Ciapaite J, van Dijk TH, Havinga R, van der Zee EA, Groen AK, Reijngoud D-J, Bakker BM & van Dijk G (2016) Whole-Body Vibration Partially Reverses Aging-Induced Increases in Visceral Adiposity and Hepatic Lipid Storage in Mice. *PLoS One* 11, e0149419.

Srere P a., Brazil H, Gonen L & Takahashi M (1963) The Citrate Condensing Enzyme of Pigeon Breast Muscle and Moth Flight Muscle. *Acta Chem. Scand.* 17 supl., 129–134.

Talmadge RJ & Roy RR (1993) Electrophoretic separation of rat skeletal muscle myosin heavy-chain isoforms. *J Appl Physiol* 75, 2337–2340.

Wolters JC, Ciapaite J, van Eunen K, Niezen-Koning KE, Matton A, Porte RJ, Horvatovich P, Bakker BM, Bischoff R & Permentier HP (2016) Translational Targeted Proteomics Profiling of Mitochondrial Energy Metabolic Pathways in Mouse and Human Samples. *J. Proteome Res.* 15, 3204–13.
